# Supplementary material for: RTK signalling promotes epithelial columnar cell shape and apical junction maintenance in human lung progenitor cells
Source: Development. 2023 Jun 1;150(11):dev201284. doi: 10.1242/dev.201284 (PMC10281517; doi:10.1242/dev.201284)
Supplement: Supplementary information [file develop-150-201284-s1.pdf]

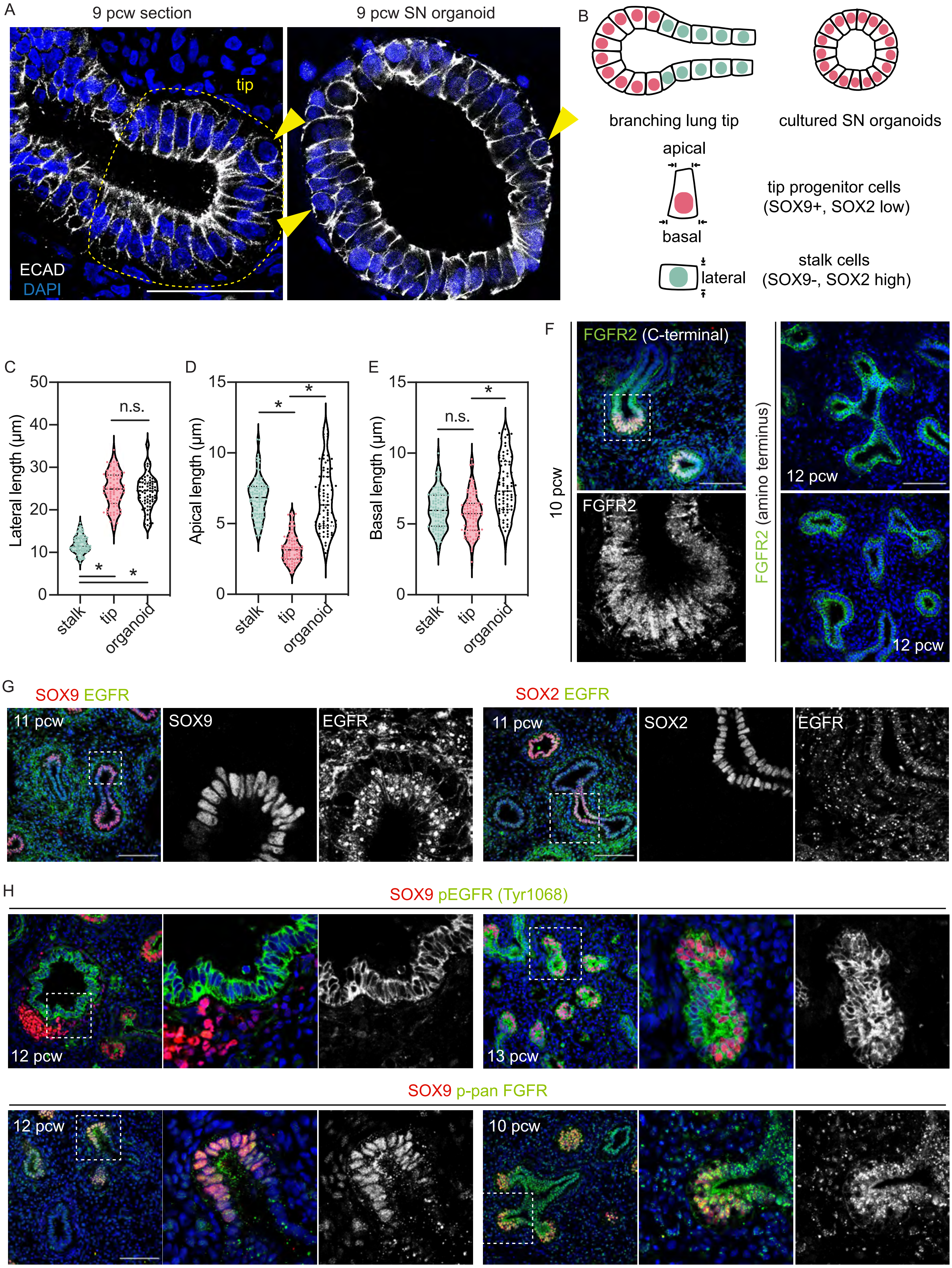

Development • Supplementary information

Fig. S1. Characterization of the epithelial cell shape and receptor expression in the pseudoglandular stage human lungs. (Related to Figure 1)

(A) Representative images showing the comparison of cell shape and arrangement of the tip epithelial cells *in vivo* and SN organoid cells *in vitro*. Yellow arrowheads show proliferating cells. (B) Diagram showing the quantitation strategy for the epithelial cells. Apical, basal and lateral length of a fully integrated cell were manually measured in ImageJ based on E-cadherin and ZO1 staining, or F-actin staining.

Measurement of the lateral (C), apical (D) and basal (E) lengths of tip progenitor cells and stalk epithelial cells *in vivo*, and SN cells of organoids. Mean±s.e.m. are shown. Blue dots show individual measurements. *$P < 0.05$ (Man-Whitney U test, N = 3 biological replicates).

(F) Expression pattern of FGFR2 (detecting amino terminus) in the early pseudoglandular lung. (G) Expression pattern of EGFR in the early pseudoglandular lung (Note the cytoplasm- and nuclear-localized staining consistent with other studies).

(H) Expression pattern of phospho-EGFR and phospho-pan FGFR in the early pseudoglandular lung (Note that the mesenchymal SOX9 seen in the left panel is developing cartilage surrounding the more proximal airways).

Scale bars = 50 µm (A); 100 µm (F, G, H).

Development • Supplementary information

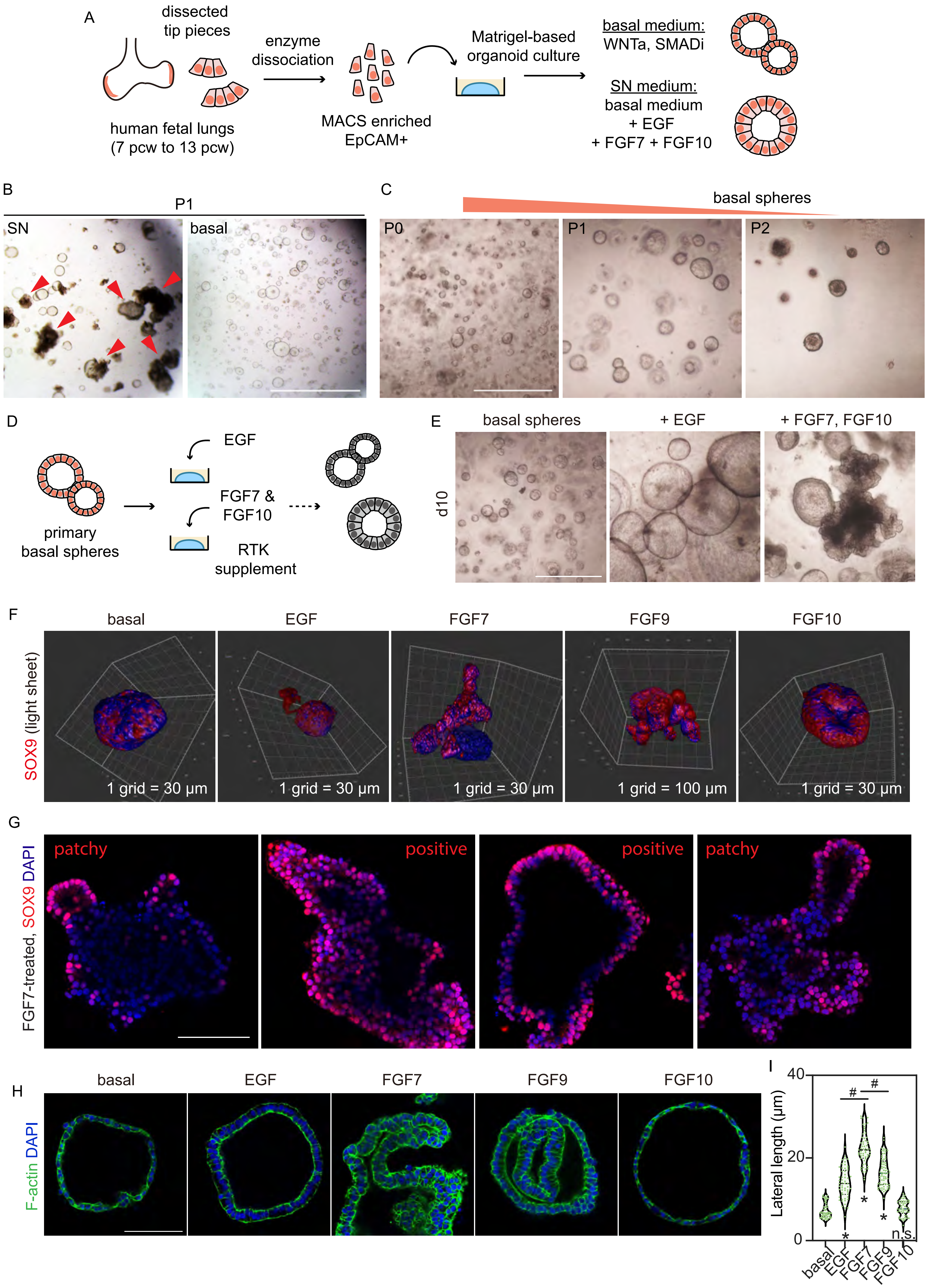

Development • Supplementary information

Fig. S2. Establishment of the basal sphere culture for RTK ligand stimulation assays. (Related to Figure 2)

(A) Experimental design` freshly-dissected lung tips were dissociated into single cells. MACS- enriched epithelial cells (5,000 per well) were seeded in Matrigel and cultured in a basal medium (RSO1,` Chir99021, Noggin, SB431542) or SN medium (basal medium plus EGF, FGF` and FGF10) and grown into 3D structures.

(B) Representative images showing organoid morphologies in the basal medium or SN medium. Red arrowheads showing budding organoids.

(C) Representative images showing basal spheres at different passages.(C

(D) Experimental design` primary basal spheres were distributed to different wells and received different R r̀ (EGF, FGF7 or FGF10) supplements for 10 to 14 days.

(E) Representative images showing organoid morphologies in different conditions.

(F) ight` sheet images (reconstructed in Arivis Vision4D) showing expression pattern of SOX9 in the d10 organoids. Related to Figure 2F.

(G) Expression pattern of SOX9 in the FGF-treated organoids. Related to Figure 2F.`

(H) F-actin (ActinGreen 488) staining showing organoid morphology and cell shape of the d10 organoids.

(I) uantitation` of lateral length of cells in each condition based on F-actin staining. Mean±s.e.m. are shown. Green dots show individual measurements. **P* < 0.05 compared to cells in the basal medium and . *P* < 0.05 compared between conditions indicated in the graph (Man-Whitney U test, N = 3 biological replicates). Scale bars = 1 mm (B, C, E); 100 µm (G, H).

Development • Supplementary information

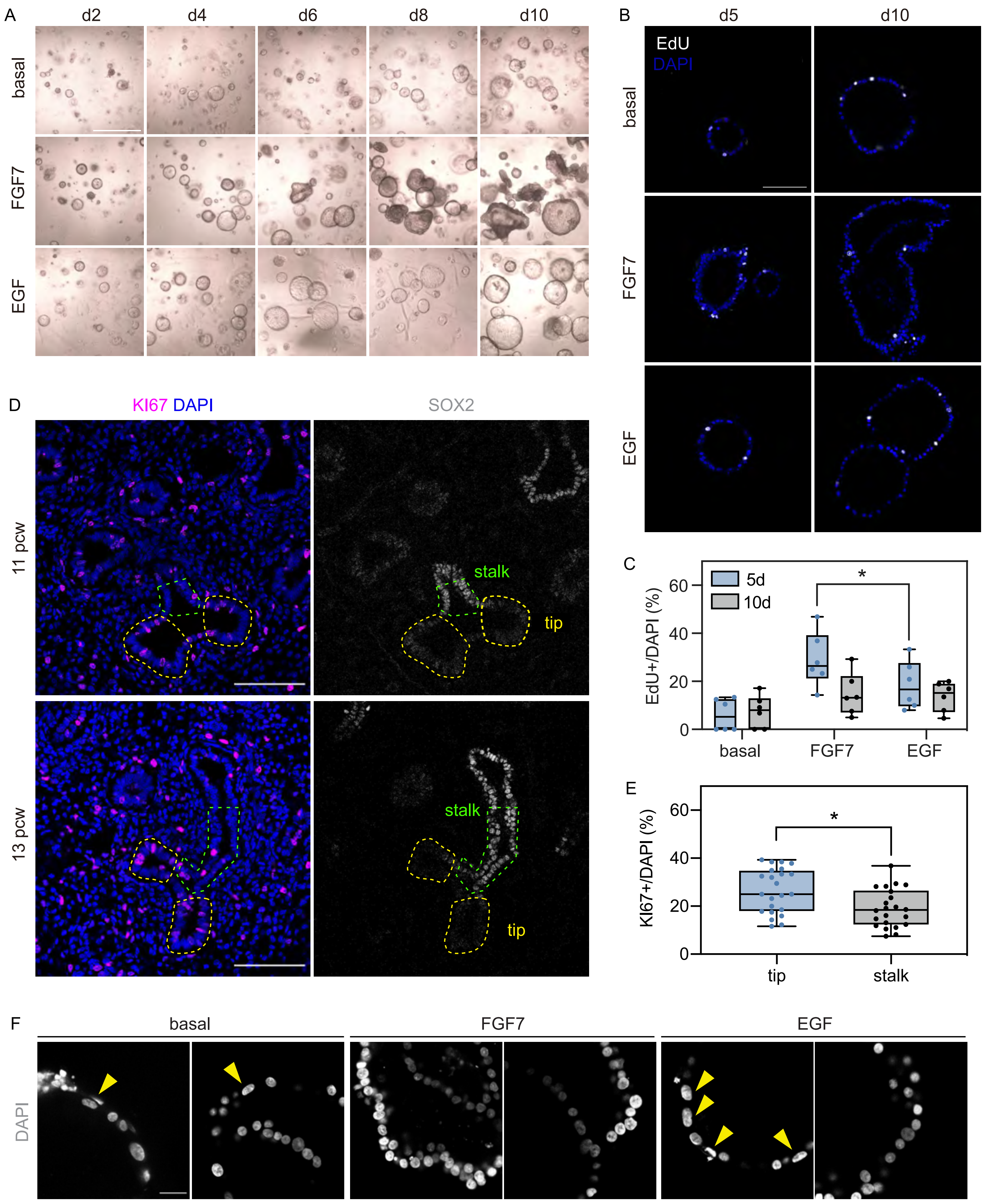

Development • Supplementary information

Fig. S3. Time-course characterization of FGF7- and EGF-treated organoids. (Related to Figure 3)
(A) Representative images showing a time-course of organoid morphologies following FGF7 or EGF stimulation.
(B) EdU assay showing proliferating cells at d5 and d10 (protocol shown in Fig. 2E).
(C) Quantitation of EdU positive cells of d5 and d10 organoids. In house ImageJ plug-in OAK (Dr. Richard Butler, available at https://github.com/gurdon-institute/OAK/tree/master/OAK) was used to score the number EdU positive cells. Mean±s.e.m. are shown. Blue and black dots show individual measurements. **P* < 0.05 (Man-Whitney U test, N = 3 biological replicates).
(D) Representative images showing cell proliferation in the early pseudoglandular human lungs. (E) Quantitation of KI67+ cells in the tip and stalk epithelia. Mean±s.e.m. are shown. Blue and black dots show individual measurements. **P* < 0.05 (Man-Whitney U test, N = 4 biological replicates).
(F) Representative images showing nuclear shape of d10 organoids. Yellow arrowheads showing flat nuclei. Related to Figure 3D and 3H.
Scale bars = 1 mm (A); 100 μm (B, D); 20 μm (F).

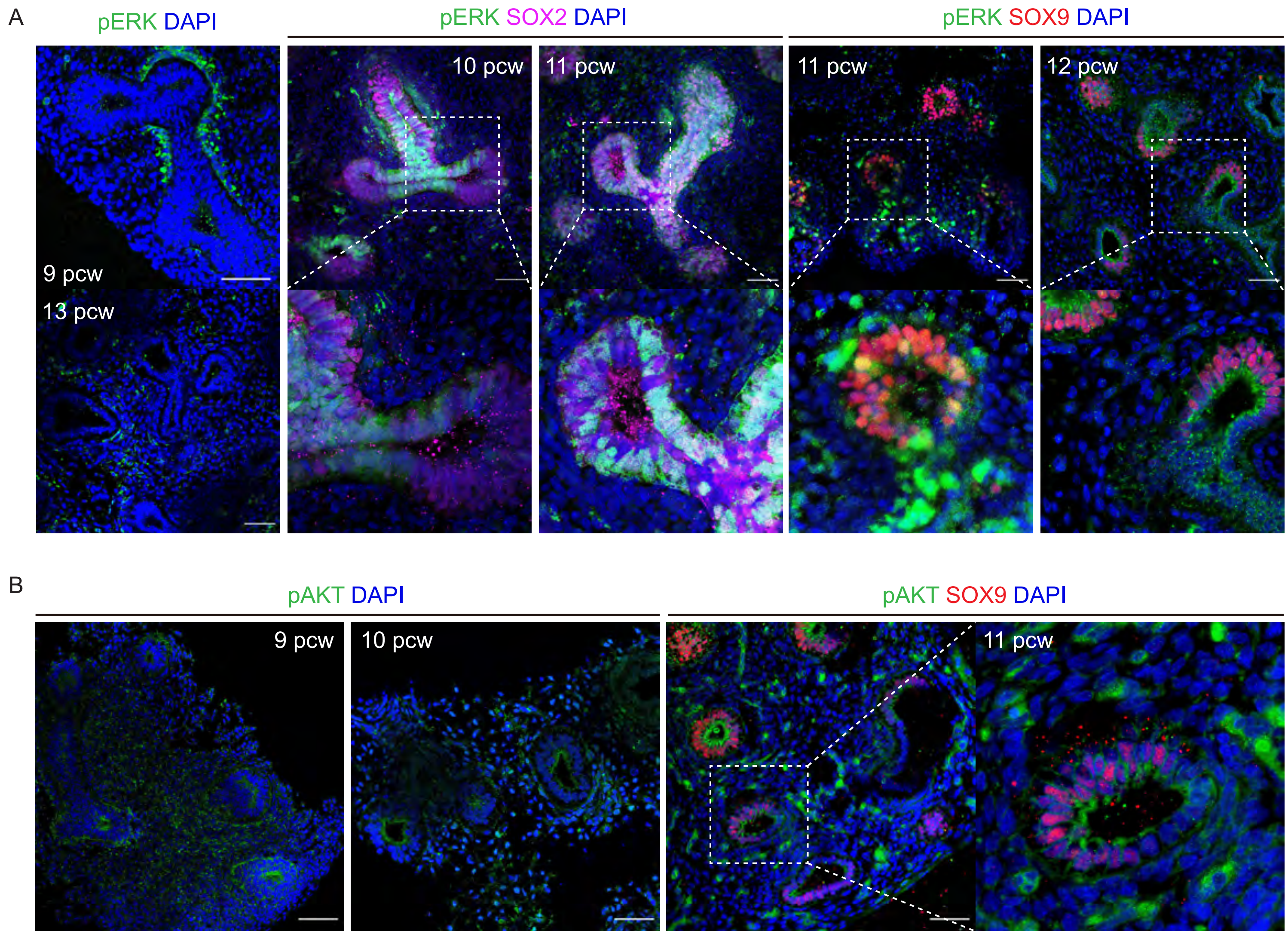

Fig. S4. Characterization of pERK and pAKT in the pseudoglandular stage human lungs. Related to Figure 4.

Expression pattern of pERK (A) and pAKT (B) in the early pseudoglandular lung. (N=6 biological replicates shown in A and 3 biological replicates in B).

Note that the staining level and pattern varies extensively from sample to sample. This is probably because these phospho-proteins are turned over very rapidly and there are differences in time between isolation and fixation of each sample which is out of the control of the laboratory.be

Scale bars = 100 µm.

Development • Supplementary information

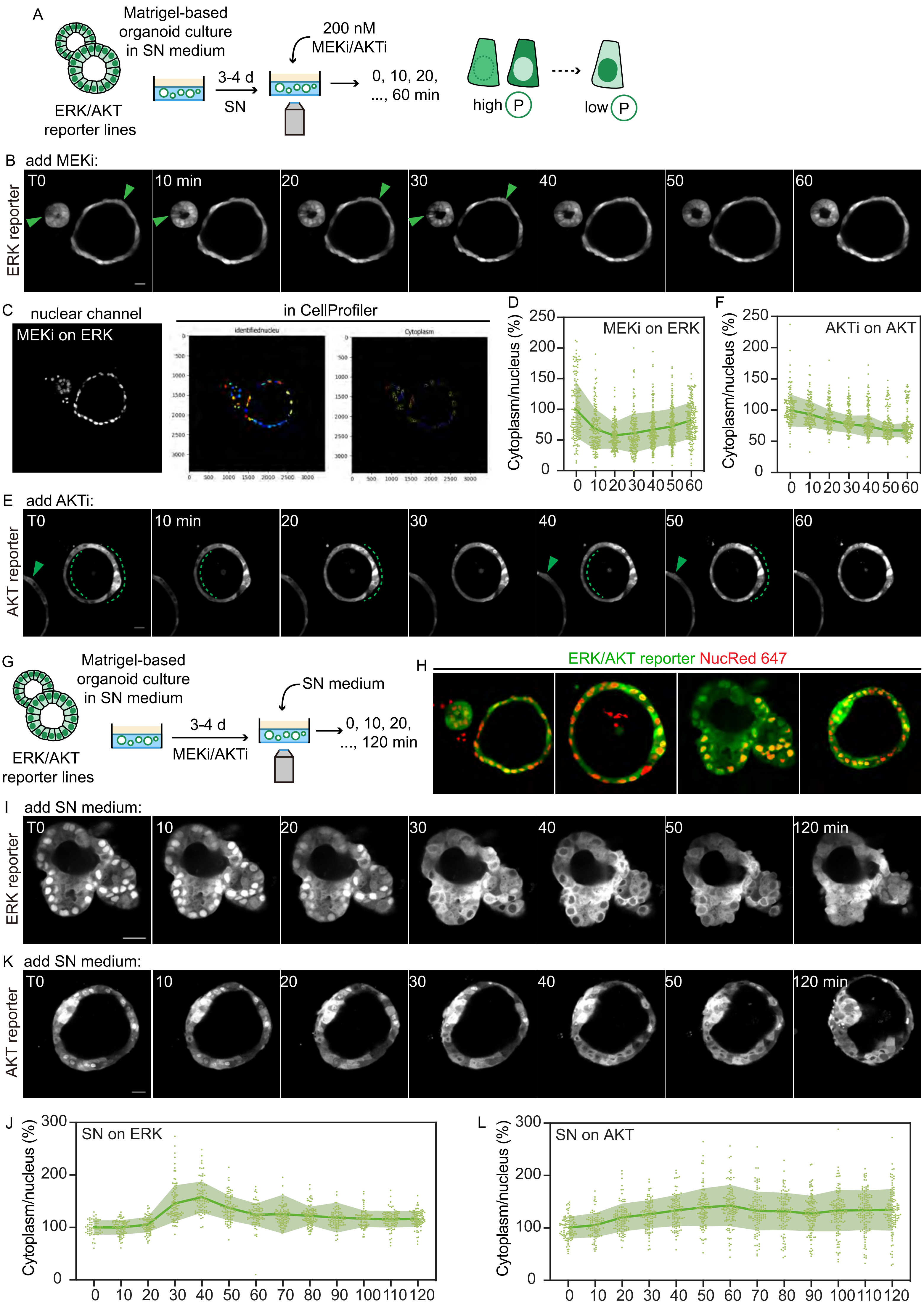

Development • Supplementary information

Fig. S5. Validation of KTR reporter live-imaging strategy in the organoid system. (Related to Figure 4)

(A) Experimental design: ERK or AKT KTR reporter organoids were transferred into a 96-well imaging plate and cultured in SN medium to recover from passaging. MEKi (PD0325901, 200 nM) or AKTi (MK2206, 200 nM) was added to the culture when live-imaging was performed (T0) and the experiment lasted for 60 min. Images of multiple stacks were taken every 10 min during the live-imaging.

(B) Representative images showing that the ERK reporter cells responded to MEK inhibition during the live-imaging. Green arrowheads show cells with clear cytoplasm-to-nucleus fluorescence translocation, indicating a reduction in ERK activity.

(C) Images showing the quantitation strategy in CellProfiler. The nuclear channel of the live- imaged organoid was segmented (as 'identifiednucleus') and the cytoplasm was identified as a grown region (5 pixels) based on the segmented nuclei. Fluorescence intensities for the identified nuclei and cytoplasm of each identified cell (from one image showing one Z plane at one time- point) were recorded.

(D) Quantitation of the ERK signal obtained as the cytoplasmic/nuclear intensity for the organoid shown in B. Each point represents one scored cell, dark green line shows the average, light green line shows the 95% confidence intervals. All cytoplasmic/nuclear intensity were normalized to the mean at T0 and shown as a percentage.

(E) Representative images showing the AKT reporter cells responding to AKT inhibition during the live-imaging. Green arrowheads and dashed line show cells with clear cytoplasm-to-nucleus fluorescence translocation indicating a reduction in AKT activity.

(F) Quantitation of the AKT signal obtained as the cytoplasmic/nuclear intensity for the organoid shown in E. Each point represents one scored cell, dark green line shows the average, light green line shows the 95% confidence intervals. All cytoplasmic/nuclear intensity were normalized to the mean at T0 and are shown as percentage.

(G) Experimental design: ERK or AKT reporter organoids in the 96-well imaging plate were treated with MEKi (PD0325901, 200 nM) or AKTi (MK2206, 200 nM) after the cells recovered from passaging. SN medium was added to the culture when live-imaging was performed (T0) and the experiment lasted for 120 min. Images of multiple stacks were taken every 10 min during the live-imaging.

(H) Representative images showing the reporter channel (green) and nuclear staining channel (NucRed 647, red) of live-imaged organoids.

Representative images showing the ERK reporter cells (I) and AKT reporter cells (K) which received SN medium during the live-imaging. Fluorescence moves from the nucleus to the cytoplasm indicating increased ERK (I) or AKT (K) activity. Quantitation of the ERK signal (J) and AKT signal (L) obtained as the cytoplasmic/ nuclear intensity for the organoids shown in H and J, respectively. Each point represents one scored cell, dark green line shows the average, light green line shows the 95% confidence intervals. All cytoplasmic/nuclear intensity were normalized to the mean at T0 and shown as percentage.

Scale bars = 20 µm (B, E, I, K).

Development • Supplementary information

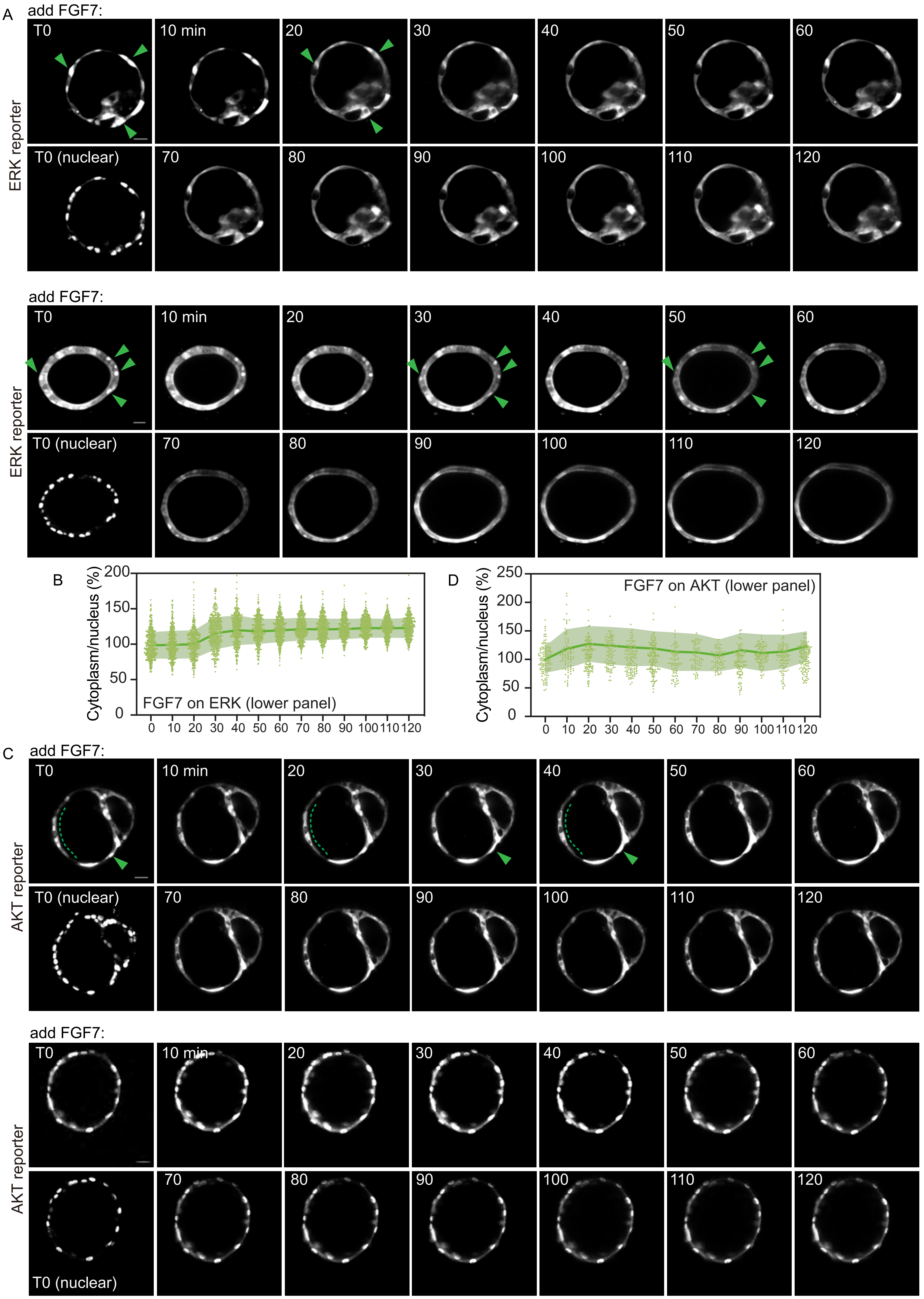

Development • Supplementary information

Fig. S6. KTR-based live-imaging showing that FGF7 treatment on MEKi- or AKTi- treated organoids restored pERK or pAKT. (Related to Figure 4)

Images showing the ERK reporter (A) or AKT reporter (C) cells following FGF7 stimulation. Green arrowheads and dotted lines indicate cells showing evident nucleus-to-cytoplasm fluorescence translocation.

(B,D) Quantitation of the ERK signal obtained as the cytoplasmic/nuclear intensity for the organoids shown in A (lower panel) and C (lower panel). Each point represents one scored cell, dark green line shows the average, light green line shows the 95% confidence intervals. All cytoplasmic/nuclear intensity were normalized to the mean at T0 and shown as percentage.

Scale bars = 20 µm.

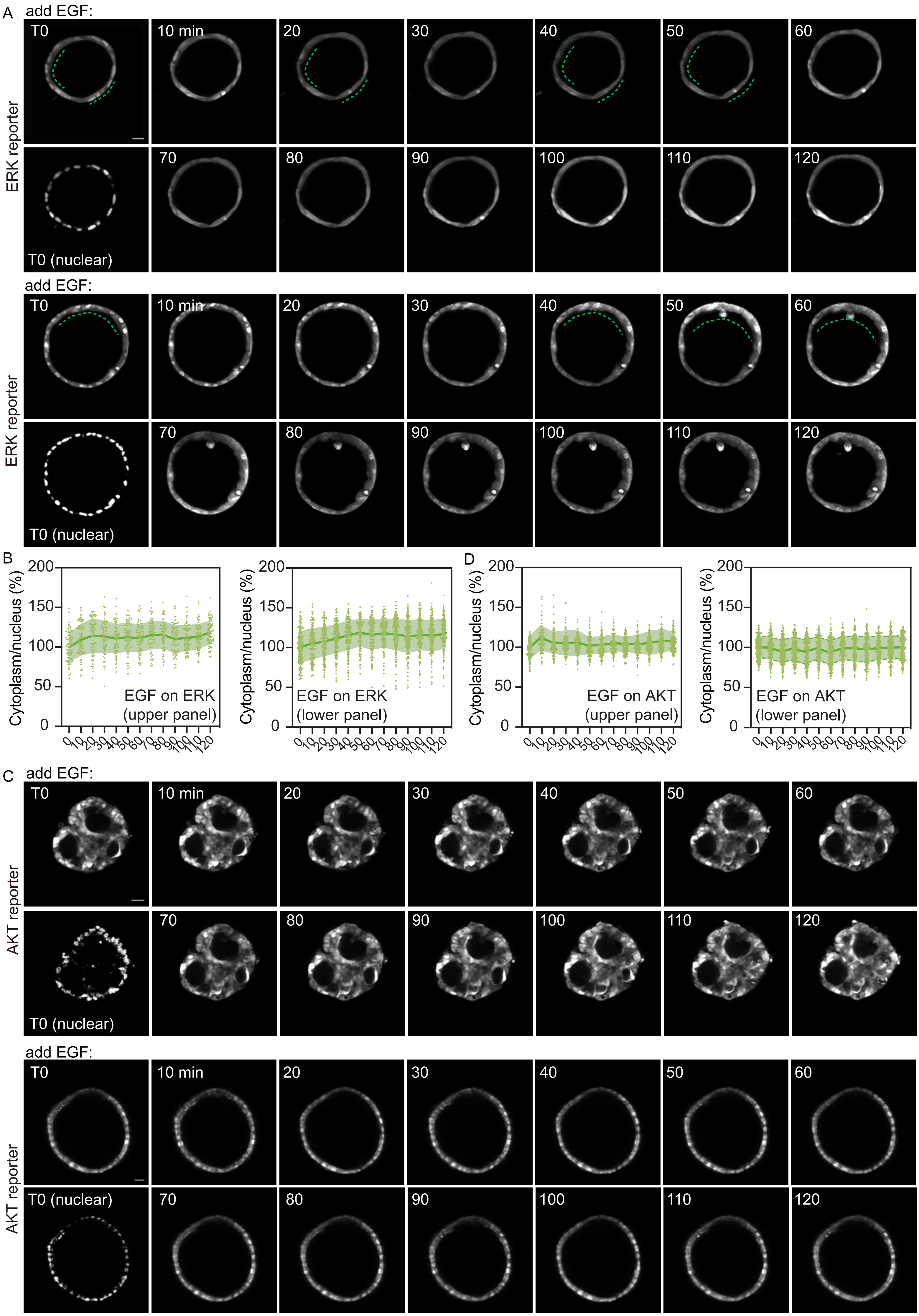
A
add EGF:
T0
10 min
20
30
40
50
60
70
80
90
100
110
120
T0 (nuclear)
ERK reporter
B
D
Cytoplasm/nucleus (%)
EGF on ERK
(upper panel)
EGF on ERK
(lower panel)
EGF on AKT
(upper panel)
EGF on AKT
(lower panel)
C
AKT reporter

Fig. S7. KTR-based live-imaging showing that EGF treatment on MEKi- or AKTi-treated organoids restored pERK, but not pAKT. (Related to Figure 4)

Images showing the ERK reporter (A) or AKT reporter (C) cells following EGF stimulation. Green arrowheads and dotted lines indicate cells showing evident nucleus-to-cytoplasm fluorescence translocation.
(B,D) Quantitation of the ERK signal obtained as the cytoplasmic/nuclear intensity for the organoids shown in A and C.
Each point represents one scored cell, dark green line shows the average, light green line shows the 95% confidence intervals. All cytoplasmic/nuclear intensity were normalized to the mean at T0 and shown as percentage.
Scale bars = 20 μm.

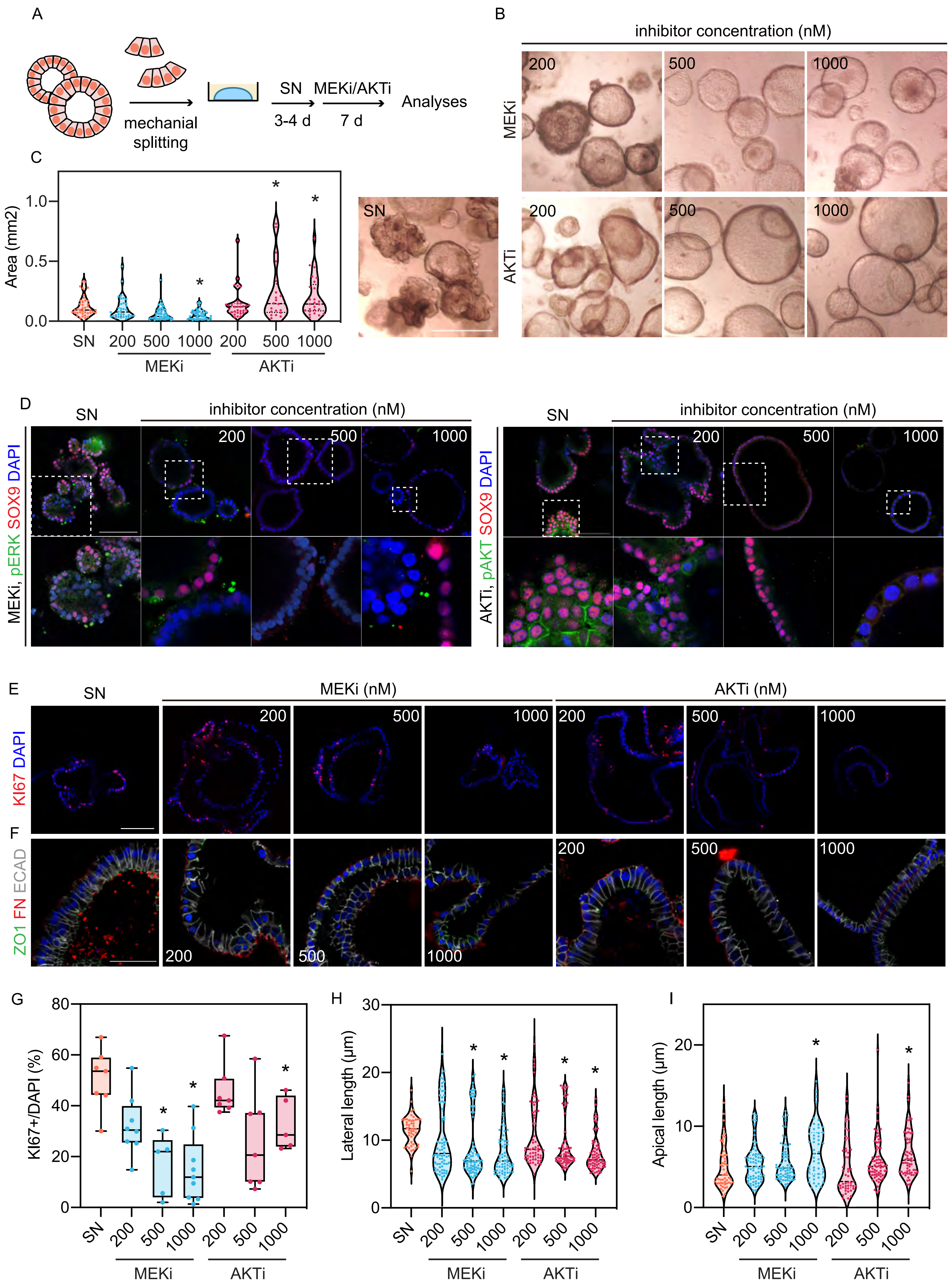

Development • Supplementary information

Fig. S8. Dose-dependent effects of MEKi and AKTi on the SN organoids. (Related to Figure 5)
(A) Experimental design: SN organoids were treated with 200, 500 or 1000 nM MEKi (PD0325901) or AKTi (MK2206) and analysed after 7 days of the inhibition.
(B) Representative images showing organoid morphology after 7 days of inhibition.
(C) Quantitation of projected area of d7 organoids by in-house Fiji plug-in (Dr. Richard Butler). Mean ±s.e.m. are shown. Coloured dots show individual measurements. *$P < 0.05$ (Man-Whitney U test, N = 3 biological replicates).
(D) Loss of SOX9 and decreased pERK or pAKT were detected with increasing inhibitor doses. (E) Representative images showing cell proliferation in organoids after 7 days of inhibition. (F) Representative images showing cell shape of organoids after 7 days of inhibition.
Quantitation of cell proliferation (G), lateral length (H) and apical length (I) of the organoids related to F. Mean±s.e.m. are shown. Coloured dots show individual measurements. *$P < 0.05$ (Man-Whitney U test, N = 3 biological replicates).
Scale bars = 1 mm (B); 100 µm (D, E); 50 µm (F).

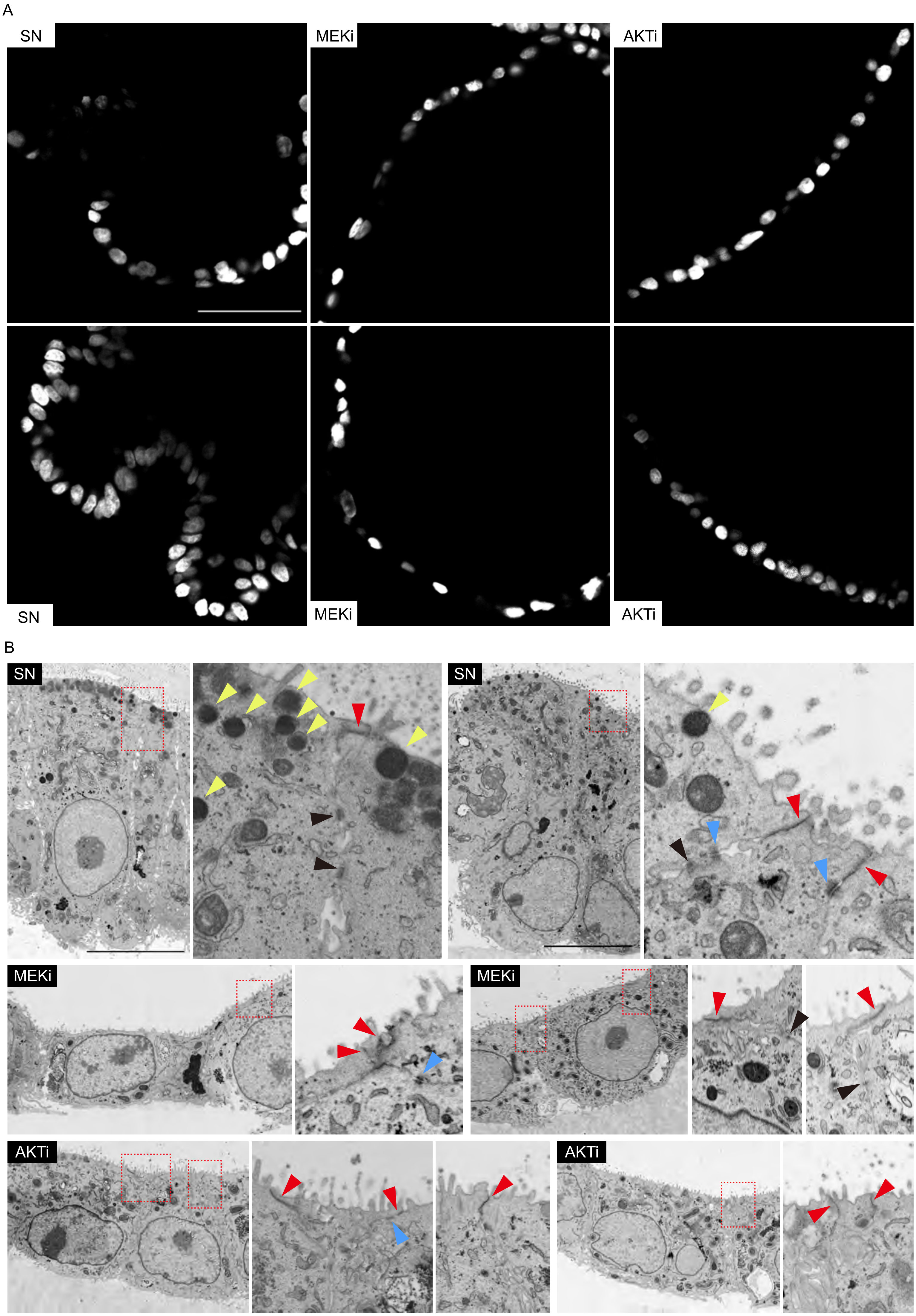

A
SN
MEKi
AKTi
SN
MEKi
AKTi
B
SN
SN
MEKi
MEKi
AKTi
AKTi

Fig. S9. Immunostaining images and SEM images showing cell nuclei, cell structure and junctions in SN, MEKi and AKTi organoids. (Related to Figure 5)

(A) Representative images showing cell nuclei of organoids after 7 days of inhibition. Scale bar = 50 µm. Related to Figure 5B and 5H.

(B) Representative SEM images showing zonula occluden/adheren (red arrowheads), occludin/claudin (blue arrowheads), adherens junction structure (black arrowheads), and vesicles (yellow arrowheads). Scale bars = 10 µm. Related to Figure 5I.

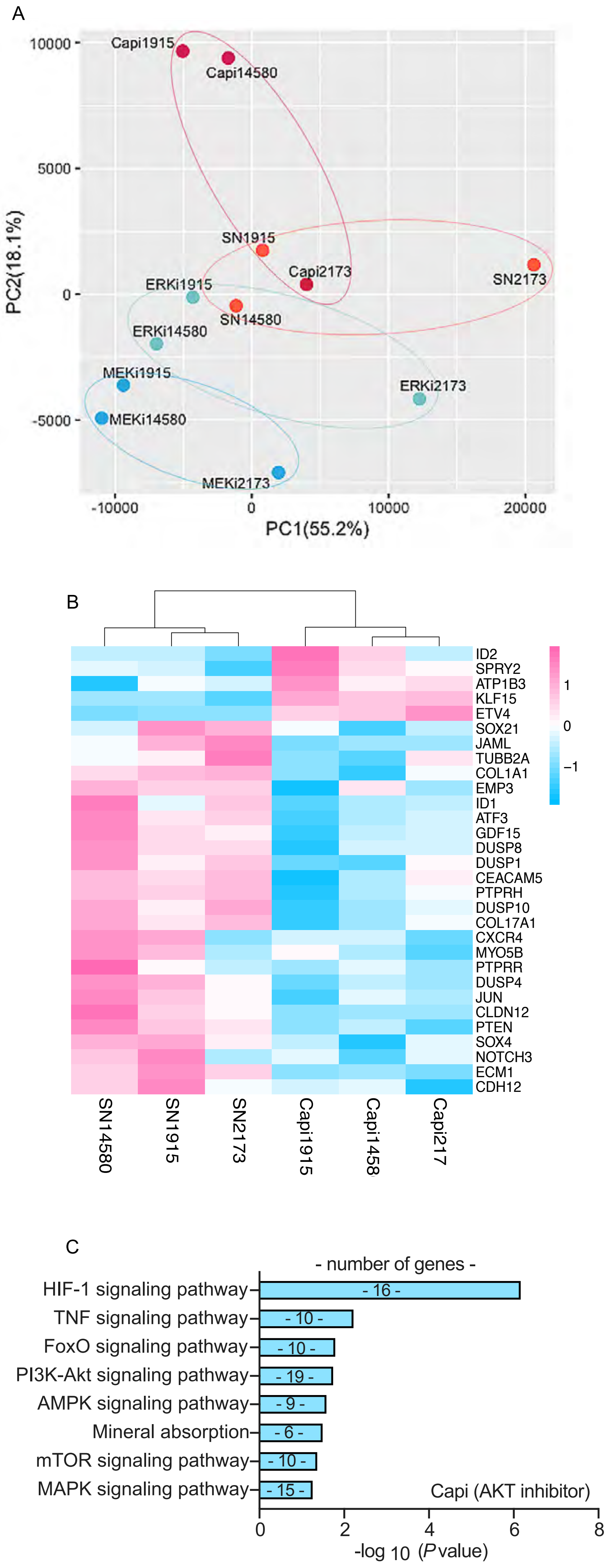

Development • Supplementary information

Fig. S10. Bulk RNA-seq results showing gene expression variation among organoids treated with different inhibitors. (Related to Figure 6)

(A) Principal component analysis of bulk RNA-seq data.

(B) Heatmap showing expression level of selected genes significantly altered in AKT-inhibited cells.

(C) KEGG pathway analysis (selected terms) of the down-regulated genes in the AKT-inhibited cells; $\log_2 FC > 1$, adjusted p value < 0.05.

Development • Supplementary information

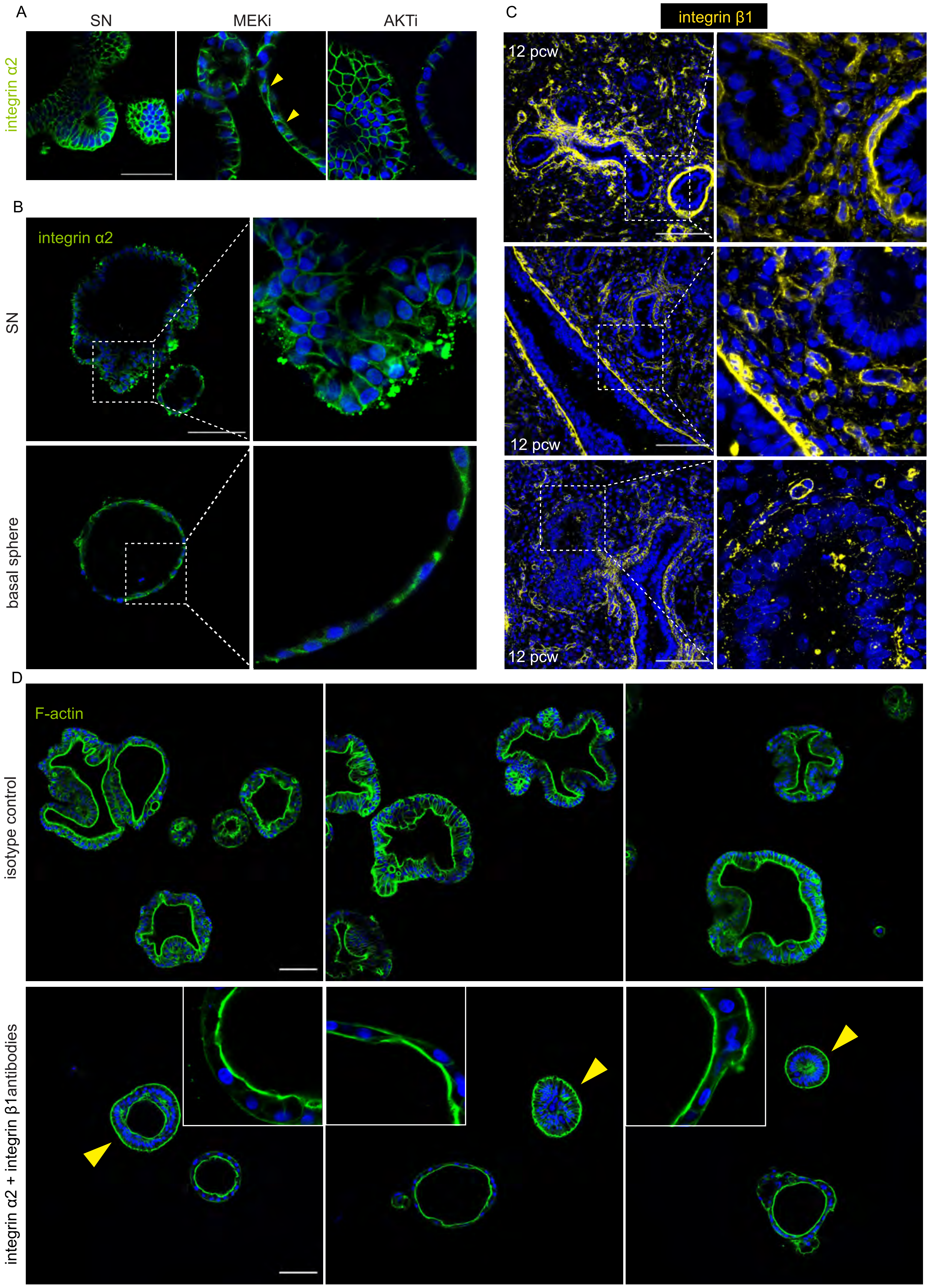

Development • Supplementary information

Fig. S11. Expression of integrins and organoid morphologies of integrin-blocked cells. (Related to Figure 6) (A) Representative images showing integrin α2 expression pattern in SN organoids and organoids treated with MEKi (PD0325901, 200 nM) or AKTi (MK2206, 200 nM) for 7 days. Yellow arrowheads show altered integrin α2.

(B) Representative images showing integrin α2 expression pattern in SN organoids and basal spheres derived from freshly-dissected epithelial tips (as in Fig. 2A) at P1.

(C) Representative images showing integrin β1 expression pattern in three 12 pcw human lungs. (D) F-actin staining showing organoid morphology, cell shape and cell polarity of organoids treated with isotype control antibody or integrin antibodies. Yellow arrowheads: organoids showing inverted apical-basal polarity. Related to Figure 6F.

Scale bars = 100 µm.

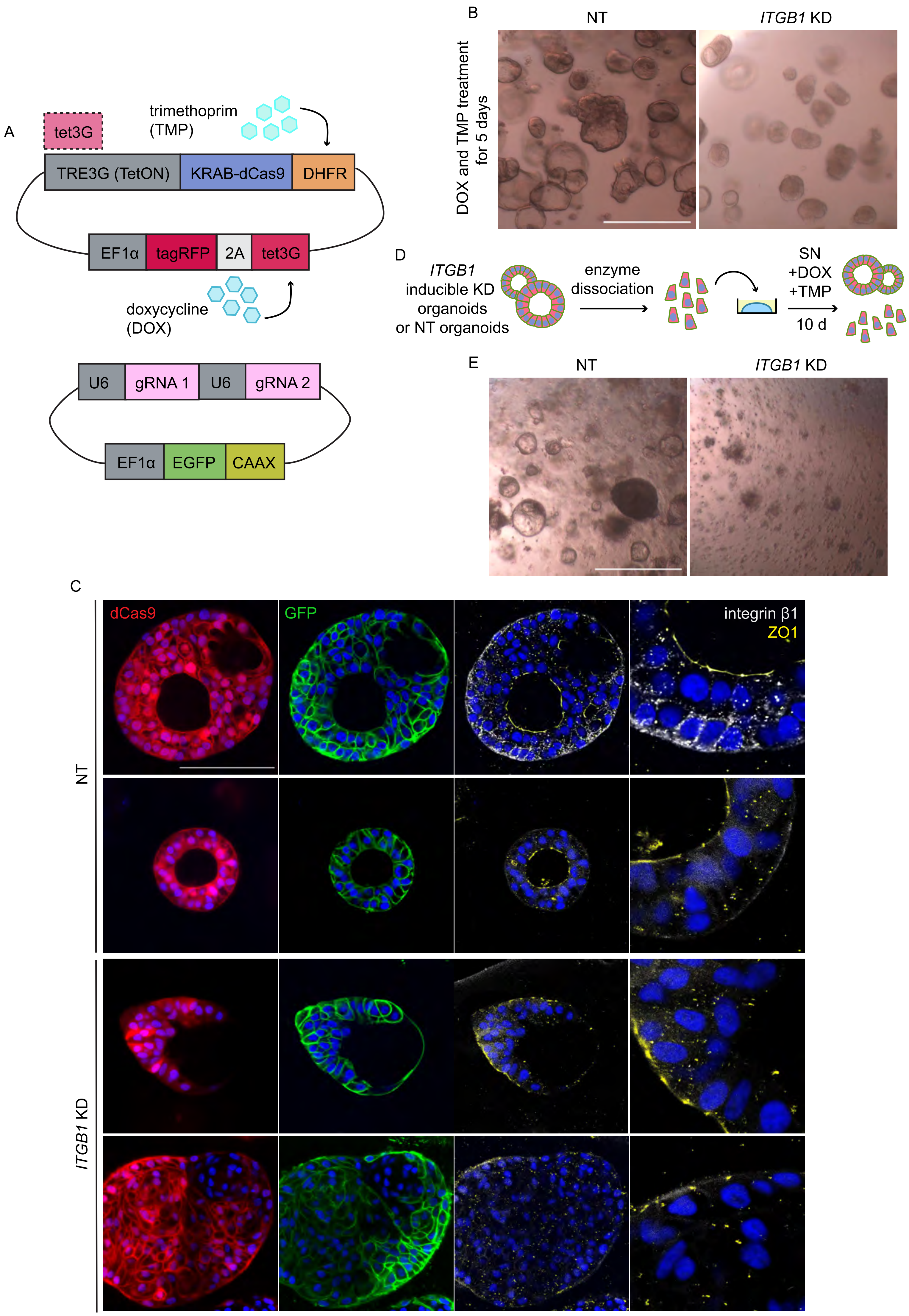

Development • Supplementary information

Fig. S12. Inducible knockdown of *ITGB1* in the organoids. (Related to Figure 6)

(A) Diagram showing the doxycycline inducible KRAB-dCas9 vector and gRNA vector.

(B) Representative images showing organoid morphology of *ITGB1*-knockdown (KD) organoid and non-targeting (NT) control after 5 days of 2 μg/ml DOX and 10 μM TMP treatment.

(C) Representative images showing organoid morphology, cell shape and polarity of *ITGB1*-KD organoids and NT control organoids after 5 days of DOX and TMP treatment.

(D) Experimental design: ITGB1-KD organoids or NT control organoids were enzymatically dissociated into single cells and seeded (10,000 per well). 2 μg/ml DOX and 10 μM TMP were added to the single cells for 10 days.

(E) Representative images showing organoid morphology of the passaged single cells after 10 days of DOX and TMP treatment.

Scale bars = 1 mm (B, E); 100 μm (C).

Development • Supplementary information

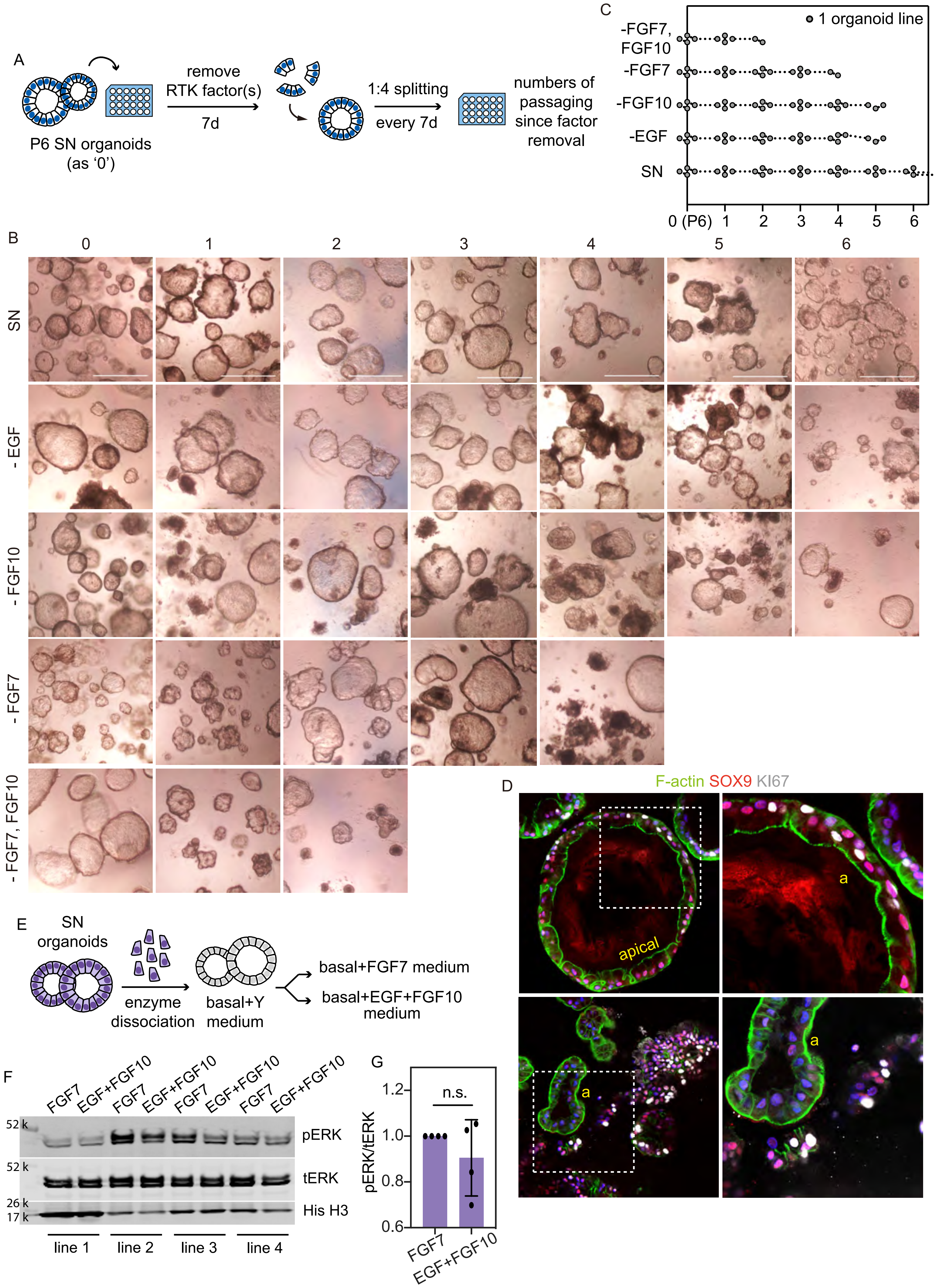
A
P6 SN organoids (as '0')
remove RTK factor(s)
7d
1:4 splitting
every 7d
numbers of passaging since factor removal
C
1 organoid line
-FGF7, FGF10
-FGF7
-FGF10
-EGF
SN
0 (P6) 1 2 3 4 5 6
B
0 1 2 3 4 5 6
SN
- EGF
- FGF10
- FGF7
- FGF7, FGF10
D
F-actin SOX9 KI67
apical
a
E
SN organoids
enzyme dissociation
basal+Y medium
basal+FGF7 medium
basal+EGF+FGF10 medium
F
FGF7 EGF+FGF10 FGF7 EGF+FGF10 FGF7 EGF+FGF10 FGF7 EGF+FGF10
52 k
pERK
52 k
tERK
26 k
17 k
His H3
line 1 line 2 line 3 line 4
G
n.s.
pERK/tERK
1.2
1.0
0.8
0.6
FGF7
EGF+FGF10

Fig. S13. FGF10 is required for the maintenance of SN organoids in vitro. (Related to Figure 7) (A) Experimental design: P6 SN organoids were cultured in media with one or more RTK factors removed from the SN medium. Surviving organoids were passaged every 7d.

(B) Representative images showing organoids cultured in different conditions.

(C) Survival plot of organoids cultured in each condition. N = 4 organoid lines (8 to 12 pcw) were tested (each point represents one surviving organoid line).

(D) Representative images showing organoid morphology and SOX9 expression of organoids in FGF10-removed condition after 5 passages.

(E) Experimental design: SN organoids were dissociated into single cells and cultured in basal medium plus 10 μM ROCK inhibitor, Y27632. The basal spheres were distributed to different wells and supplied with 100 ng/ml FGF7, or 50 ng/ml EGF+100ng/ml FGF10 for 10 days.

(F) pERK levels of FGF7-organoids and organoids in combined EGF+FGF10 treatment.

(G) Mean and s.e.m. of pERK level (normalized to tERK, His H3 as loading control). Black dots show individual measurements. FGF7 exerted similar level of ERK activation (shown as pERK) as EGF plus FGF10 did. Student's t test was used for significance evaluation, n.s. = not significant. Scale bars = 1 mm (B); 100 μm (D).

Table S1. RNAseq data in tab delimited format. (Related to Figures 6 and S10).

Click here to download Table S1

Development • Supplementary information

Table S2. Primary antibodies

	Supplier	Cat. No.	Host species	Dilution
SOX9	R&D Systems	AF3075	Goat	1:200
SOX2	Bio-Techne	AF2018	Goat	1:200
ZO1	ThermoFisher Scientific	40-2200	Rabbit	1:200
E-cadherin	ThermoFisher Scientific	13-1900	Rat	1:1000
Fibronectin	R&D Systems	AF1918	Sheep	1:400
FGFR2 (C-terminal)	Santa Cruz	SC-122	Rabbit	1:200
FGFR2 (amino terminal)	Cell Signaling Technologies	23323S	Rabbit	1:200
phospho-pan FGFR	Cell Signaling Technologies	3476S	Mouse	1:100
phospho-EGFR	Cell Signaling Technologies	3777S	Rabbit	1:200
phospho-ERK	Cell Signaling Technologies	4370S	Rabbit	1:200
phospho-AKT	Cell Signaling Technologies	4060S	Rabbit	1:200
integrin α2	AbCam	ab181548	Rabbit	1:400
integrin β1	AbCam	ab30394	Mouse	1:400
KI67	BD	550609	Mouse	1:400
CD31	AbCam	ab9498	Mouse	1:400
GFP	AbCam	ab13970	Chicken	1:200

Table S3. Secondary antibodies

	Supplier	Cat. No.	Host species	Dilution
Donkey anti-mouse 488	ThermoFisher Scientific	A21202	Mouse	1:2000
Donkey anti-rabbit 488	ThermoFisher Scientific	A21206	Rabbit	1:2000
Donkey anti-goat 488	ThermoFisher Scientific	A11055	Goat	1:2000
Donkey anti-rat 488	ThermoFisher Scientific	A21208	Rat	1:2000
Donkey anti-chicken 488	Jackson Immune	703-545-155	Chicken	1:2000
Donkey anti-mouse 594	ThermoFisher Scientific	A21203	Mouse	1:2000
Donkey anti-rabbit 594	ThermoFisher Scientific	A21207	Rabbit	1:2000
Donkey anti-goat 594	ThermoFisher Scientific	A11058	Goat	1:2000
Donkey anti-sheep 594	Jackson Immune	713-585-147	Sheep	1:1000
Donkey anti-goat 647	ThermoFisher Scientific	A21447	Goat	1:2000
Donkey anti-rat 647	Jackson Immune	712-605-153	Rat	1:2000

Development • Supplementary information

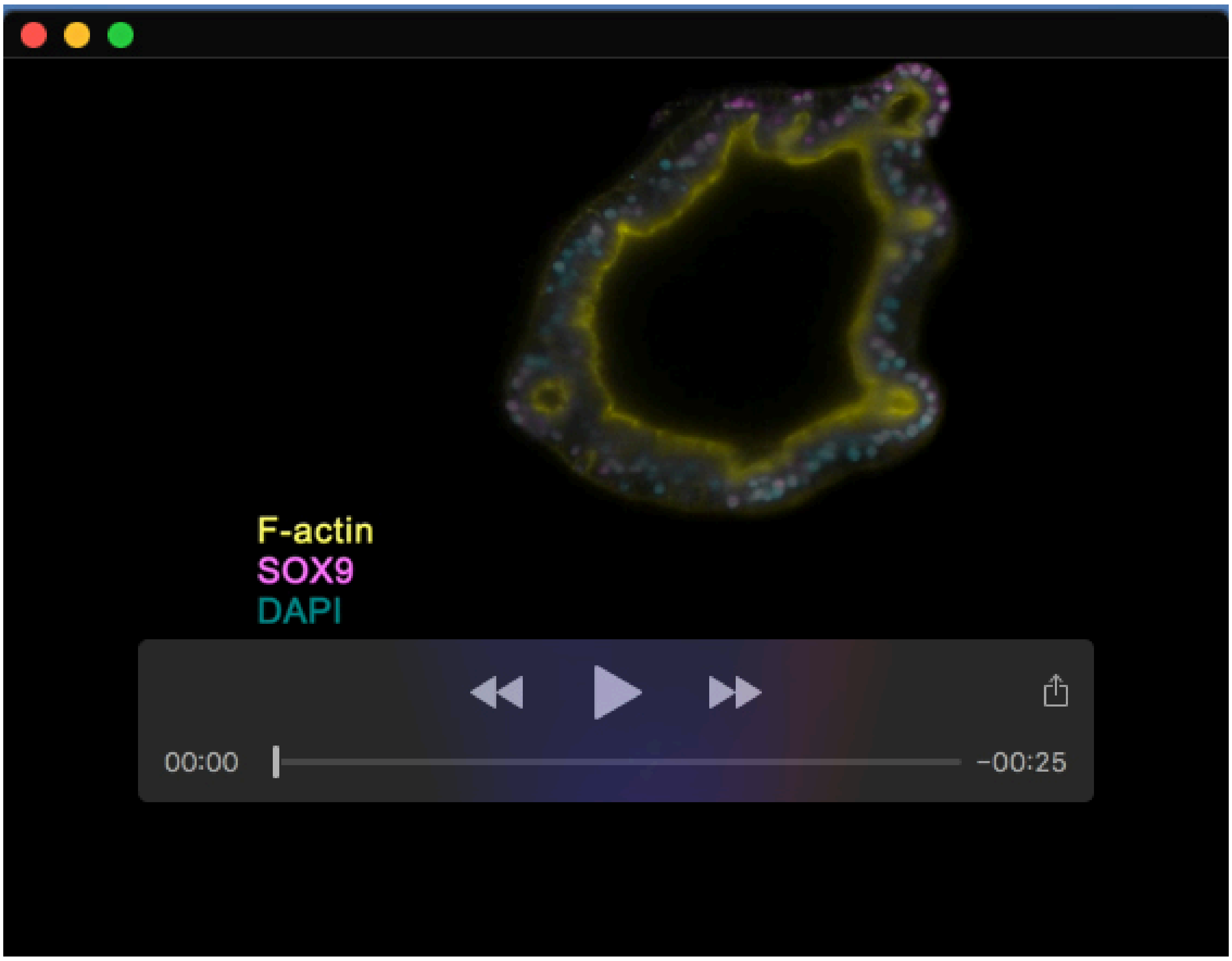

Movie 1. 3D reconstructed self-renewing organoid to show cell arrangement. Stained with F-actin (yellow), SOX9 (magenta) and DAPI (blue).
