# Supplementary material for: RTK signalling promotes epithelial columnar cell shape and apical junction maintenance in human lung progenitor cells
Source: Development. 2023 Jun 1;150(11):dev201284. doi: 10.1242/dev.201284 (PMC10281517; doi:10.1242/dev.201284)
Supplement: Supplementary file 1 [file develop-150-201284_review_history.pdf]

RTK signalling promotes epithelial columnar cell shape and apical junction maintenance in human lung progenitor cells

Shuyu Liu, Dawei Sun, Richard Butler and Emma L. Rawlins
DOI: 10.1242/dev.201284

Editor: James Wells

Review timeline
Original submission: 9 September 2022
Editorial decision: 19 October 2022
First revision received: 21 April 2023
Accepted: 10 May 2023

Original submission

First decision letter

MS ID#: DEVELOP/2022/201284

MS TITLE: RTK signalling promotes epithelial columnar cell shape and apical junction maintenance in human lung progenitor cells

AUTHORS: Shuyu Liu, Dawei Sun, Richard Butler, and Emma L Rawlins

I have now received all the referees' reports on the above manuscript, and have reached a decision. The referees' comments are appended below, or you can access them online: please go to BenchPressand click on the 'Manuscripts with Decisions' queue in the Author Area.

As you will see, the referees express considerable interest in your work, but have some significant criticisms and recommend a substantial revision of your manuscript before we can consider publication. If you are able to revise the manuscript along the lines suggested, which may involve further experiments, I will be happy receive a revised version of the manuscript. Your revised paper will be re-reviewed by one or more of the original referees, and acceptance of your manuscript will depend on your addressing satisfactorily the reviewers' major concerns. Please also note that Development will normally permit only one round of major revision. If it would be helpful, you are welcome to contact us to discuss your revision in greater detail. Please send us a point-by-point response indicating your plans for addressing the refereeâ€™s comments, and we will look over this and provide further guidance.

Please attend to all of the reviewers' comments and ensure that you clearly highlight all changes made in the revised manuscript. Please avoid using 'Tracked changes' in Word files as these are lost in PDF conversion. I should be grateful if you would also provide a point-by-point response detailing how you have dealt with the points raised by the reviewers in the 'Response to Reviewers' box. If you do not agree with any of their criticisms or suggestions please explain clearly why this is so.

© 2023. Published by The Company of Biologists under the terms of the Creative Commons Attribution License (https://creativecommons.org/licenses/by/4.0/).

Reviewer 1

Advance summary and potential significance to field

Liu et al. showed that FGF7, FGF10 and EGF activated the ERK and AKT pathways to promote organoid budding and proliferation of primary human embryonic tip epithelial cells. Their findings further supported that FGF7 among the three RTK inputs had the greatest potential in promoting cell proliferation and maintaining columnar cell shape. Although FGF10 itself showed minimal potential, combination with FGF7 or EGF presented concerted function in organoid maintenance through increasing downstream signaling activities. The authors further demonstrated that Fgf7 modulate tip cell shape in part through integrin (a2 and B1). These findings are very interesting, complementary with the group's recent findings by Sun et al., providing a molecular mechanism involved in lung budding and cell shaping in early human lung development.

Comments for the author

A few minor questions need to be considered.

1. Line 175-177. Can the authors postulate why at d10 the difference is lost between basal vs. fgf7 or EGF group? Given there is difference at d5, basal group must catch up with fgf7/EGF group during d5-d10. Is possible that fgf7/Egf groups slow down in expansion during the period. What could be the possible explanation for this?
2. FigS1G, nice EGFR staining showed enriched in the cell surface. How about the strong punctate staining in the nuclei? It seems the nuclear signals are also present in the surrounding mesenchyme. The authors may want to make a note whether it is specific or non-specific staining.
3. Integrin perturbation is very interesting. The authors justified and showed the expression of a2. How about ß1 expression in the developing lungs?

Reviewer 2

Advance summary and potential significance to field

In this paper, Liu et al. revealed the distinct roles of FGF7, EGF and FGF10 in cell shape maintenance of human lung tip progenitors. Using a human fetal lung tip ex vivo culture system, the authors successfully determined that FGF7 activates MAPK/ERK and PI3K/AKT signaling and is sufficient to promote columnar cell shape in primary tip progenitors. By contrast, EGF was found to be less potent because of its inefficiency in activating AKT. The combination of EGF and FGF10 was sufficient to maintain human tip progenitor in long-term culture. The authors also revealed that integrin signaling is a key pathway downstream of MAPK/ERK. Disruption of integrin alters the polarity, cell adhesion and tight junction assembly of the tip progenitors.

Whereas the mechanisms of lung tip development and branching morphogenesis have been investigated extensively in mice, we still lack knowledge on the same mechanisms in humans. This report demonstrates important insights into human respiratory biology, particularly highlighting that the function of RTK signaling and ligand specificity differ from those in mice. I feel that the manuscript is suitable for publication in the journal pending the incorporation of additional data and responding to the specific comments listed below to improve their manuscript.

Comments for the author

The authors cultured human lung tips with AKT inhibitors and found that the AKT pathway is involved in the regulation of cell morphology downstream of Fgf7. However, genes related to cell adhesion and the cytoskeleton do not appear to be altered in Fig. S10. The authors should explain how AKT activation affects budding morphology.

The authors propose that the combination of EGF and FGF10 induces budding morphology by increasing the amount of pERK at the lung tips. If this is the case, the FGF7-induced budding may

© 2023. Published by The Company of Biologists under the terms of the Creative Commons Attribution License (https://creativecommons.org/licenses/by/4.0/).

also be explained by the amount of pERK. To clarify this relationship, the protein levels of pERK with EGF, FGF10, and FGF7 should be compared with Western Blots.

In Fig. S2H, the columnar morphology of FGF7- and FGF9-treated cells should be quantified as in Fig. 1C.

In Fig. S2F, the size of the organoid supplied with EGF looks smaller than that with basal medium, which is a different result from other figures. The authors should explain this inconsistency.

Reviewer 3

Advance summary and potential significance to field

See below.

Comments for the author

The authors used a tissue-derived organoid system to study the cellular mechanisms, such as cell shape, in human lung development. They focused on FGFs and EGF that are required in their culture media and examined known downstream signaling pathways including MAPK/ERK, PI3K/AKT, and integrins. While the body of work presented is extensive, their conclusions seem limited in impact and significance and are somewhat predictable from the literature and their other publications including “SOX9 maintains human foetal lung tip progenitor state by enhancing WNT and RTK signalling”. As detailed below, this is due to the difficulty to causally link signaling to cellular changes and to provide a coherent explanation for conflicting data, as well as inclusion of unconvincing data.

Are the cellular phenotypes, especially the columnar cell shape, the direct target of RTK signaling? Alternatively, they could be a secondary consequence of, for example, cell density, that depends on organoid growth, which is expected to be most robust in the complete media. Several experiments would alleviate this concern. First, individual organoids in the same condition are of different size and can be “branching” or cystic (e.g. Fig. 2B-Fgf7/9 treatments). Do both small and large organoids have the columnar cell shape? Cells in “branching” organoids are more likely be caught at an oblique section angle and thus appear more columnar. Second, are the cellular phenotypes cell-autonomous, as predicted by their conclusions? A mixed culture to generate organoids made of normal and knockdown cells would address this. Third, to conclude integrin signaling as a mediator of RTK signaling controlling cell shape, rather than an independent pathway disrupting cell adhesion and polarity requires a rescue experiment by re-expressing integrins in the presence of RTK signaling inhibition.

Does knocking down or blocking Itga2 alone affect cell shape? Broadening to Itgb1, a partner for the majority of alpha integrins, when their RNA-seq data implicated specific integrins, weakens their conclusions to the generic “integrin signaling”, which is predictably important. It does not help with the specificity and significance to describe integrins are regulated by MAP/ERK and also regulate MAP/ERK.

Another example of insufficient understanding of conflicting data is that they spent much of the manuscript on FGF7, contrasting it to FGF10 and EGF and the former’s role in the mouse lung. This comparison was made less impactful by the suggestion at the end of the manuscript that FGF10 and EGF are somehow involved for cell shape maintenance. Relatedly, they used two organoid culture systems (self-renewing and basal sphere) and two dissociation methods (mechanical and enzymatic) interchangeably, although there are differences including the mentioned conflicting data on FGF10 and EGF. Does gene function depend on culture conditions? Which condition recapitulates gene function in vivo?

A number of immunostaining images are unconvincing. For example, Fig. 4A, 4B and 4E. Why is the FGFR2 signal nuclear in Fig. 1G? Fig. 1H needs single channel images for p-pan FGFR and p-EGFR like 1G. If stronger data is not available, the gap in evidence needs to be stated. Line 116 says “removing EGF did not cause a striking morphology change (Fig. 1F)”, but the images look

© 2023. Published by The Company of Biologists under the terms of the Creative Commons Attribution License (https://creativecommons.org/licenses/by/4.0/).

otherwise to this reviewer. Line 329: “integrin protein was altered (Fig. S11A)”. Could this expression pattern simply be a result of cell shape change and not altered integrin? Fig. 4K’ is so variable that it needs statistical testing. Fig. 6B: how are the genes selected? If based on differential expression between treatments, clustering by treatment is expected by design. It should be top variable genes across samples without grouping by treatments.

Minor points:
Line 30: extra period or typo somewhere.
Line 65: “its role in the development” extra “the”
Line 179: “a high level of proliferation was required for branching in vivo and organoid budding in vitro”. Should be correlated without evidence for requirement.
Line 291: The method here should be transmission electron microscopy (TEM).
Line 297: “apical surface…was stretched” what does stretched mean in this context? How is stretch classified in TEM?
Line 327: “high abundance in organoids” the figure referenced is of tissue, not organoids.
Line 360: fig. S11C does not appear to be related to the “integrin antibody blocked organoids”
Fig. 1B: missing DAPI label Fig 1D: typo in “passaging”
Fig. 3D: first panel is missing outline of closeup area Fig. 5I: label style between panels does not match (AKTi is black).
Fig. S2F says patchy while the main text says partial

First revision

<u>Author response to reviewers' comments</u>

We would like to thank the editor for being willing to wait for the revised version of this manuscript. In the last few months the first author has completed her PhD, performed revision experiments for this manuscript, visited her family in China for the first time in 4 years, and moved to the USA to begin a postdoctoral position. This somewhat delayed our ability to put the revised version of the manuscript together.

Reviewer 1 Advance Summary and Potential Significance to Field:
Liu et al. showed that FGF7, FGF10 and EGF activated the ERK and AKT pathways to promote organoid budding and proliferation of primary human embryonic tip epithelial cells. Their findings further supported that FGF7 among the three RTK inputs had the greatest potential in promoting cell proliferation and maintaining columnar cell shape. Although FGF10 itself showed minimal potential, combination with FGF7 or EGF presented concerted function in organoid maintenance through increasing downstream signaling activities. The authors further demonstrated that Fgf7 modulate tip cell shape in part through integrin (a2 and ß1). These findings are very interesting, complementary with the group’s recent findings by Sun et al., providing a molecular mechanism involved in lung budding and cell shaping in early human lung development.

Reviewer 1 Comments for the Author:
A few minor questions need to be considered.

1. Line 175-177. Can the authors postulate why at d10 the difference is lost between basal vs. fgf7 or EGF group?
Given there is difference at d5, basal group must catch up with fgf7/EGF group during d5-d10. Is possible that fgf7/Egf groups slow down in expansion during the period. What could be the possible explanation for this?

Fig. S3C shows the quantitation of EdU positive cells in the basal spheres, and FGF7- and EGF-treated organoids after 5 or 10 days of ligand treatment. As the reviewer points out there were significant differences in proliferation in response to FGF7 and EGF at d5, but not at d10. We speculate that there is negative feed-back on proliferation rates in the system, likely mediated paracrine secretion of factors from neighbouring organoids as cell/organoid density increases in the highly proliferative conditions. We have added a statement to this effect to the text: Whereas

© 2023. Published by The Company of Biologists under the terms of the Creative Commons Attribution License (https://creativecommons.org/licenses/by/4.0/).

differences were no longer observable at d10, possibly due to paracrine secretion of negative regulators of proliferation as cell/organoid density increases (Fig. S3B,C).

2.FigS1G, nice EGFR staining showed enriched in the cell surface. How about the strong punctate staining in the nuclei? It seems the nuclear signals are also present in the surrounding mesenchyme. The authors may want to make a note whether it is specific or non-specific staining.
Fig. S1G shows the staining of EGFR on two 11 pcw human embryonic lungs, which localized in both the cell surfaceand cytoplasm/nuclei of the epithelial cells and mesenchyme. We reproducibly observed this expression pattern in pseudoglandular samples. Interestingly, there was even more punctate staining in the nuclei in a 22 pcw sample (Reviewer Figure 1). Given that EGFR can be nuclear-localized and previous studies have shown convincing nuclear and cytoplasmic EGFR (see Gururaj et al., 2013 PMID: 23250739 ; Li et al., 2009 PMID: 19684613 ; Liccardi et al., 2011 PMID: 21266349 for examples), we conclude that the EGFR staining shown in Fig. S1G is specific. We thank the Reviewer for pointing this out and have now made a note in the figure legend.

Reviewer Figure 1: Expression pattern of EGFR in a 22 pcw lung. Scale bars = 100 µm.

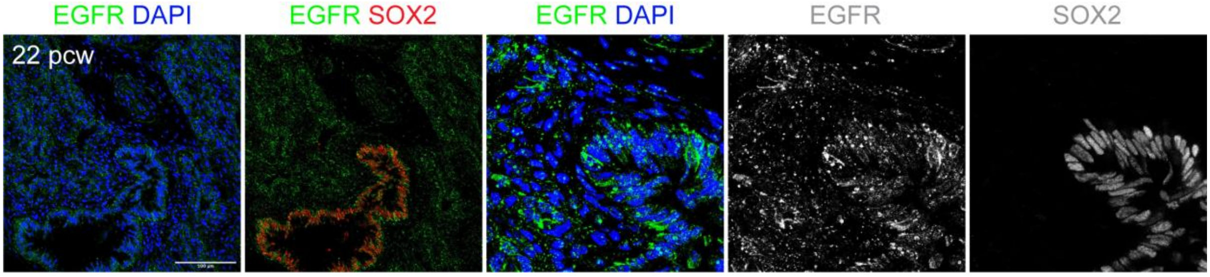

3.Integrin perturbation is very interesting. The authors justified and showed the expression of a2. How about ß1 expression in the developing lungs?
We agree with the Reviewer that perturbing integrin signalling in the tip epithelial cells is an interesting experiment following our RNA-seq findings. Expression of integrin β1 in the branching human lungs (three different 12 pcw samples) is now shown in Fig. S11 (Fig. S11 C) and described in the results section.

Reviewer 2 Advance Summary and Potential Significance to Field: In this paper, Liu et al. revealed the distinct roles of FGF7, EGF and FGF10 in cell shape maintenance of human lung tip progenitors. Using a human fetal lung tip ex vivo culture system, the authors successfully determined that FGF7 activates MAPK/ERK and PI3K/AKT signaling and is sufficient to promote columnar cell shape in primary tip progenitors. By contrast, EGF was found to be less potent because of its inefficiency in activating AKT. The combination of EGF and FGF10 was sufficient to maintain human tip progenitor in long-term culture. The authors also revealed that integrin signaling is a key pathway downstream of MAPK/ERK. Disruption of integrin alters the polarity, cell adhesion and tight junction assembly of the tip progenitors. Whereas the mechanisms of lung tip development and branching morphogenesis have been investigated extensively in mice, we still lack knowledge on the same mechanisms in humans. This report demonstrates important insights into human respiratory biology, particularly highlighting that the function of RTK signaling and ligand specificity differ from those in mice. I feel that the manuscript is suitable for publication in the journal pending the incorporation of additional data and responding to the specific comments listed below to improve their manuscript.

Reviewer 2 Comments for the Author:
The authors cultured human lung tips with AKT inhibitors and found that the AKT pathway is involved in the regulation of cell morphology downstream of Fgf7. However, genes related to cell adhesion and the cytoskeleton do not appear to be altered in Fig. S10. The authors should explain how AKT activation affects budding morphology.
We thank the Reviewer for this question. In fact, we found that genes such as *COL1A1*, *COL17A1*, *MYO5B*, *ECM1* and *CDH12* were significantly downregulated in AKT-inhibition organoids (Fig. S10B). Therefore, we consider it likely that AKT signalling-mediates cell adhesion and cytoskeleton organisation in the tip epithelial cells. We have now made this clearer in the results section:

© 2023. Published by The Company of Biologists under the terms of the Creative Commons Attribution License (https://creativecommons.org/licenses/by/4.0/).

"We identified > 180 differentially expressed genes (DEGs) between AKT-inhibited cells and SN cells, including genes associated with the cytoskeleton and cell adhesion such as, *COL1A1*, *COL17A1*, *MYO5B*, *ECM1* and *CDH12* (Fig. S10B, File. S1, log2FC > 1, *P* < 0.05)."

The authors propose that the combination of EGF and FGF10 induces budding morphology by increasing the amount of pERK at the lung tips. If this is the case, the FGF7-induced budding may also be explained by the amount of pERK. To clarify this relationship, the protein levels of pERK with EGF, FGF10, and FGF7 should be compared with Western Blots.

We agree that FGF7-induced budding and the maintenance of columnar cell shape are likely partially explained by ERK activation and the amount of pERK. We have now compared pERK levels of tip epithelial cells receiving FGF7 single ligand treatment, and EGF plus FGF10 dual ligand treatment (Figure S13E-G). Western blotting data collected from four organoid lines (derived from four different biological samples) show that the two conditions resulted in similar pERK levels (Students' *t* test), although there was biological variation. This observation further substantiates our finding that EGF and FGF10 exerted combinatorial effects on the tip epithelial cells *in vitro*.

In Fig. S2H, the columnar morphology of FGF7- and FGF9-treated cells should be quantified as in Fig. 1C.
Thank you for pointing it out. We have now quantified the lateral length of the cells based on F-actin staining and added the results in Fig. S2I.

In Fig. S2F, the size of the organoid supplied with EGF looks smaller than that with basal medium, which is a different result from other figures. The authors should explain this inconsistency.
We agree that EGF organoid in Fig. S2F appeared to be smaller than the basal spheres. This is an unfortunate technical artefact of mounting samples for the Zeiss Z1 lightsheet within a capillary. We found that large and spherical organoids (including many of the EGF-treated organoids) were broken or deformed during the mounting process. As a result, the light sheet-based imaging was slightly more biased to spherical organoids that were relatively small. This is now noted in the legend to Figure 3A which is the first time that Lightsheet microscopy images are shown.

The size distribution of the organoids imaged within their wells following different ligand treatments is in Figure 2B,D. This shows that a range of organoid sizes are present in all conditions, including that EGF-treated organoids can be smaller than basal organoids.

Reviewer 3 Comments for the Author:

The authors used a tissue-derived organoid system to study the cellular mechanisms, such as cell shape, in human lung development. They focused on FGFs and EGF that are required in their culture media and examined known downstream signaling pathways including MAPK/ERK, PI3K/AKT, and integrins. While the body of work presented is extensive, their conclusions seem limited in impact and significance and are somewhat predictable from the literature and their other publications including "SOX9 maintains human foetal lung tip progenitor state by enhancing WNT and RTK signalling". As detailed below, this is due to the difficulty to causally link signaling to cellular changes and to provide a coherent explanation for conflicting data, as well as inclusion of unconvincing data.

Are the cellular phenotypes, especially the columnar cell shape, the direct target of RTK signaling? Alternatively, they could be a secondary consequence of, for example, cell density, that depends on organoid growth, which is expected to be most robust in the complete media. Several experiments would alleviate this concern.
The chemical inhibition experiments we did suggest that the columnar cell shape was regulated by ERK and AKT signalling (Figure 5). Based on these observations, we cannot conclude that the cellular phenotypes are a direct target of RTK signalling, but it is highly likely that they are the result of RTK inputs.

First, individual organoids in the same condition are of different size and can be "branching" or cystic (e.g. Fig. 2B-Fgf7/9 treatments). Do both small and large organoids have the columnar cell shape? Cells in "branching" organoids are more likely be caught at an oblique section angle and

© 2023. Published by The Company of Biologists under the terms of the Creative Commons Attribution License (https://creativecommons.org/licenses/by/4.0/).

thus appear more columnar.
Please note that in the manuscript, we use ‘budding’ to describe organoids that displayed non-spherical morphology, not ‘branching’. We do not observe clear branching events in our organoid system, and are not claiming to do so.

We agree with the Reviewer that there is organoid size variance in all organoid conditions. This is shown in all the bright field images in our manuscript, and quantitated. In our self-renewal condition at 4 to 5 days after passaging, both small and large organoids already possess columnar cell shape regardless of their morphology (i.e., budding or spherical).

It is important to note that for all the organoid staining shown in this manuscript, we performed whole-mount immunostaining and obtained z stack confocal images to minimize artefact and misjudgement in image analysis. Additionally, organoids for cell shape quantitation were stained with basal (fibronectin) and apical (ZO1) markers to assist with orientation, and only cells where the full lateral length of the cell could be scored were measured (Figure 1B,C). This manner of analysis, and the self-renewal medium data where cell shape is independent of organoid morphology, makes it highly unlikely that the cell shape effects observed are due to the capturing of cells at an oblique angle in budding organoids.

Second, are the cellular phenotypes cell-autonomous, as predicted by their conclusions? A mixed culture to generate organoids made of normal and knockdown cells would address this.
As suggested by the Reviewer, we have now performed an organoid-based mosaic analysis by mixing and culturing *FGFR2* knockdown cells and wildtype cells. This first required establishment of *FGFR2* knock-down organoids (Reviewer Figure 2).

Unfortunately the *FGFR2* knock down was not particularly efficient (~47% knockdown at protein level, with 37% variation between biological replicates), even after testing 8 gRNAs (Reviewer Figure 2). We previously observed (Figure 4C,D) that ERK and AKT were active at low levels even in organoids grown in basal medium, and the difficulty in obtaining the knock-down lines may reflect a role for FGFR2 in cell survival. Alternatively, we may have just been unlucky. Regardless, we did the mosaic analysis as the Reviewer suggested using two organoid lines.

Shown in Reviewer Figure 3, mixed culture of *FGFR2* KD and WT cells resulted in mosaic organoids. *FGFR2*-KD cells in the mixed culture were highly varied in cell shape, whereas WT cells generally display cuboidal to columnar shape. This suggests that the phenotypes are indeed cell autonomous. However, this was a technically challenging experiment.

The aggregated organoids did not always show clear ECAD staining on lateral membranes (even between wildtype cells). We are unwilling to analyse these data further and have not included the results in the manuscript.

<u>We do not make a strong conclusion about cell autonomy in the manuscript, and cannot exclude that some/all effects of the RTK signalling we observe are non-cell autonomous.</u>

Reviewer Figure 2: Inducible knockdown of *FGFR2* in the lung tip epithelial organoids.

(A) Diagram showing the structure of doxycycline induced KRAB-dCas9 vector and gRNA vector. See Sun et al., 2021, http://doi.org/10.7554/eLife.67886 for the CRISPRi protocol
(B) Experimental design: serial lentivirus transduction to generate cells expressing KRAB-dCas9 with two gRNAs targeting *FGFR2*, or non-targeting (NT) control gRNA. Purified KRAB-dCas9$^+$gRNAs$^+$ cells were cultured into organoids and treated with 2 μg/ml doxycycline (DOX) and 10 μM trimethoprim (TMP) for 5 days.
(C) Representative images showing organoid morphology of *FGFR2*-knockdown (KD) organoid and non-targeting control after 5 days of DOX and TMP treatment.
(D) qRT-PCR showing FGFR2 downregulation by *FGFR2* gRNAs compared to NT organoids. Mean and s.e.m. are shown. Coloured dots show individual measurements. *$P < 0.05$ (student’s *t* test, N = 3).
(E) Representative flow cytometry results for validation of *FGFR2*-KD at protein level. Knockdown efficiency is shown as mean ± s.e.m., N = 3. Note that the same *FGFR2* gRNAs vector could exert

© 2023. Published by The Company of Biologists under the terms of the Creative Commons Attribution License (https://creativecommons.org/licenses/by/4.0/).

very different KD efficiency depending on the organoid line, average knockdown is 47%, but the s.e.m. is 37%; a typical experiment is shown in the plot.

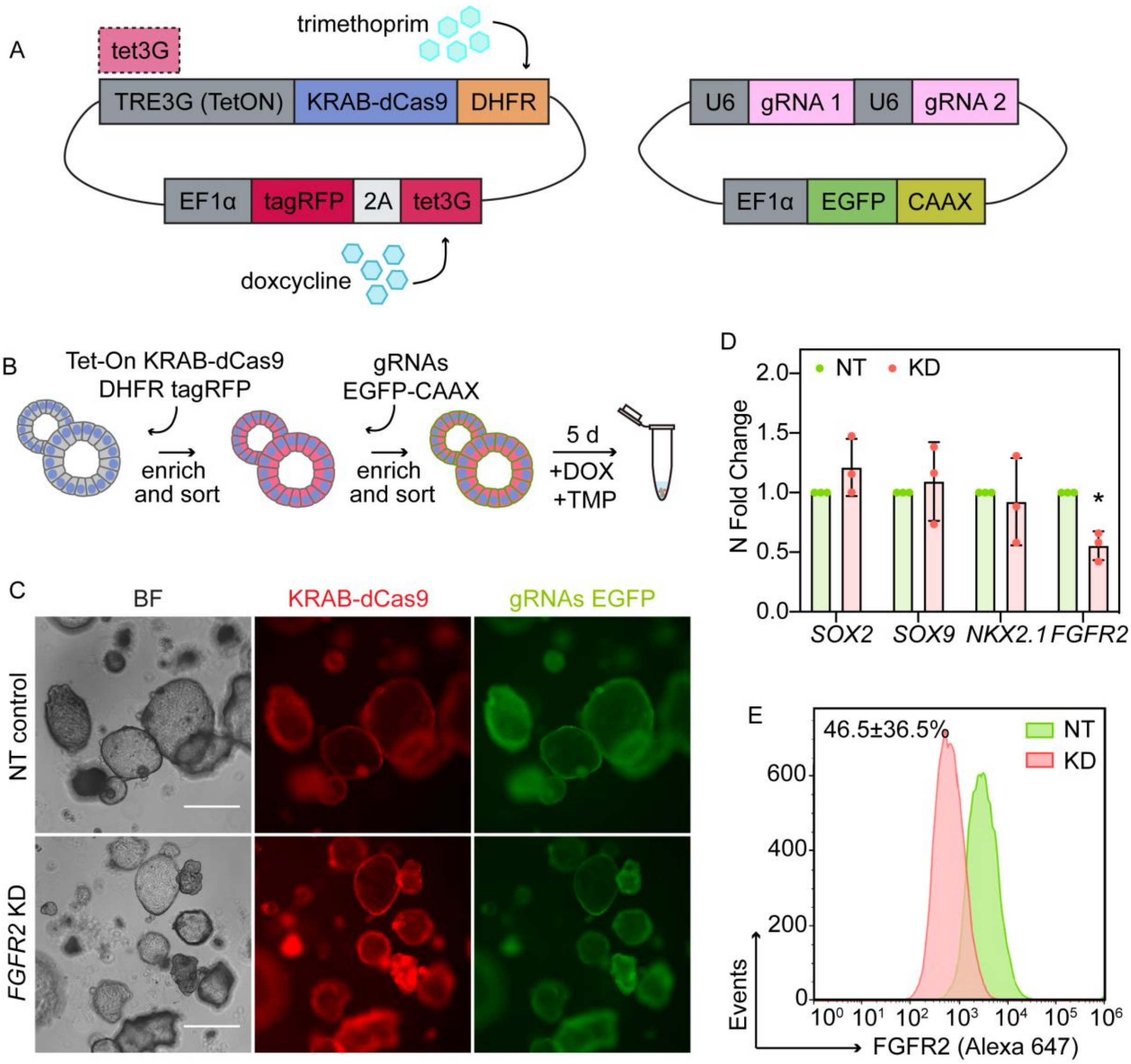

Reviewer Figure 3: Mosaic analysis of cell autonomy using *FGFR2*-KD cells.
(A) Experimental design: *FGFR2*-KD organoid and WT organoids were dissociated into single cells, mixed and cultured in suspension for 24 hours in aggrewell plates. Cell aggregates were seeded in Matrigel and cultured in SN plus DOX and TMP for 10 days. Organoids were then fixed and whole-mount stained *in situ*, i.e., the organoids were not recovered from Matrigel.
(B) Representative images showing organoid morphology and cell shape in mosaic organoids from two organoid lines. Cells co-expressing red and green are the knock-down cells. Scale bars = 50 µm.

© 2023. Published by The Company of Biologists under the terms of the Creative Commons Attribution License (https://creativecommons.org/licenses/by/4.0/).

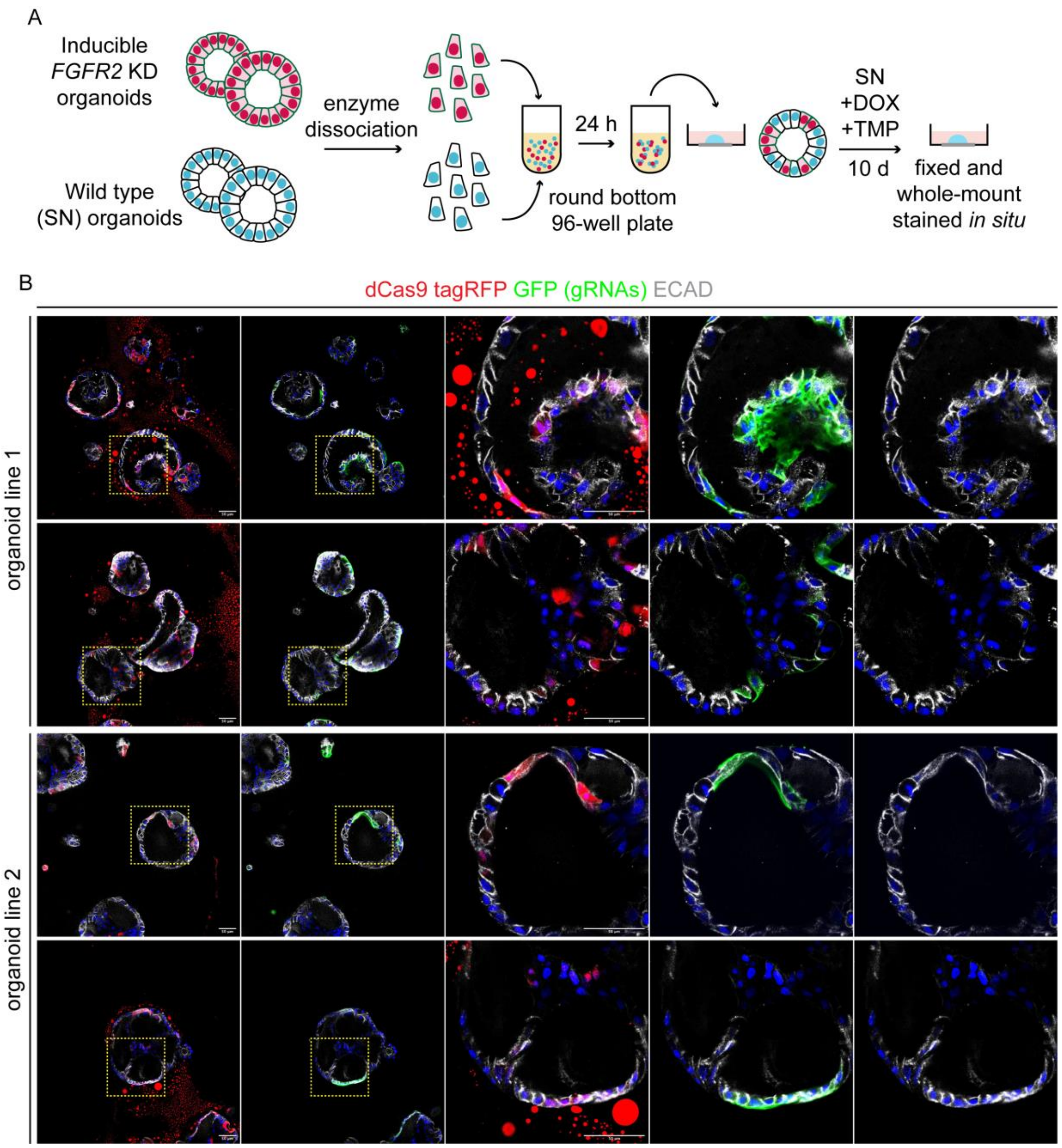

Third, to conclude integrin signaling as a mediator of RTK signaling controlling cell shape, rather than an independent pathway disrupting cell adhesion and polarity, requires a rescue experiment by re-expressing integrins in the presence of RTK signaling inhibition.

We thank the Reviewer for the comment. As suggested, we have now performed rescue experiments on the organoids with two strategies:

1. overexpressing ITGB1 genetically;

2. using $MnCl_2$ to enhance integrin-mediated cell-matrix adhesion (Bazzoni et al., 1995 PMID: 7592728).

(We did not include Pyrintegrin, a commercialized integrin activator, as it was shown to enhance phosphorylation of FGFR and EGFR (Xu et al., 2010 PMID: 20406903)).

Shown in author Reviewer Figure 4, we constructed a doxycycline-inducible *ITGB1* overexpression vector, which contains the *ITGB1* coding region (CDS) under the control of a TetOn promoter. After adding dox for 4 days, *ITGB1* was upregulated, validated by qRT-PCR. Other assessed genes, *SOX2*,

© 2023. Published by The Company of Biologists under the terms of the Creative Commons Attribution License (https://creativecommons.org/licenses/by/4.0/).

SOX9 and *ITGA2*, were not affected.
We also validated a phospho-focal adhesion kinase antibody on human embryonic lung cryosections, and used the antibody to illustrate integrin-mediated focal adhesion activities on organoids. Compared to the controls, *ITGB1* overexpression organoids exhibited both cell membrane- and some cytoplasmic-localized pFAK, implying slightly elevated focal adhesion activity and function of the over-expressed protein (Reviewer Figure 4C).
To assess whether integrin signalling could act independently from the RTK signalling, we cultured control and ITGB1 overexpression single tip epithelial cells in a basal+ medium, which did not contain any exogenous RTK ligands. The single cells grew into basal spheres, which typically were small and spherical and consisted of cuboidal cells suggesting disrupted cell junctions. We then induced *ITGB1* overexpression by adding dox to the basal spheres. After 14 days of overexpression (dox replenished every other day), we did not observe obvious organoid morphology or cell shape change after overexpressing *ITGB1*. These data suggest that integrin signalling does not work independently from the RTK signalling pathways.

These integrin OE data have not been included in the manuscript (which is already very data-heavy), but they could be added at the editor's discretion. The weakness we see with the overexpression experiment is that it is suggestive of integrin functioning downstream of RTK signalling, but does completely test the hypothesis. To test the hypothesis more thoroughly would have had to assay the effects of many other integrin family members and/or complexes which is extremely interesting but beyond the scope of the current project.

Reviewer Figure 4: Overexpressing *ITGB1* on the tip organoids could not recover cell shape disruption resulting from RTK signalling deficiency.
(A) Diagram showing the structure of doxycycline (dox) inducible *ITGB1* coding sequence (CDS) overexpression vector.
(B) qRT-PCR showing *ITGB1* upregulation by dox inducible overexpression for 4 days compared to SN control. Mean± s.d. are shown. Coloured dots show individual measurements. N = 2 biological replicates.
(C) Representative images showing focal adhesion kinase activities (pFAK: rabbit 1:100; abcam, ab81298) in *ITGB1* overexpression organoids. Scale bars = 100 μm. (C') Representative images showing pFAK expression and validating antibody staining in the developing human lung. Scale bar = 100 μm.
(D) Experimental design: *ITGB1* overexpression organoids were dissociated into single cells, seeded in Matrigel and cultured in a basal+ medium (RSPO1, Chir99021, Noggin, SB431542, Y27632) to form basal spheres. The basal spheres were treated with dox for 14 days to induce *ITGB1* overexpression.
(E) Representative images showing cell shape (illustrated by E-cadherin staining) in *ITGB1* overexpression (+ dox) organoids and SN control (- dox) from two organoids lines. Scale bars = 100 μm.
(F) Quantitation of cell lateral length from two organoid lines. Mean±s.d. are shown.

© 2023. Published by The Company of Biologists under the terms of the Creative Commons Attribution License (https://creativecommons.org/licenses/by/4.0/).

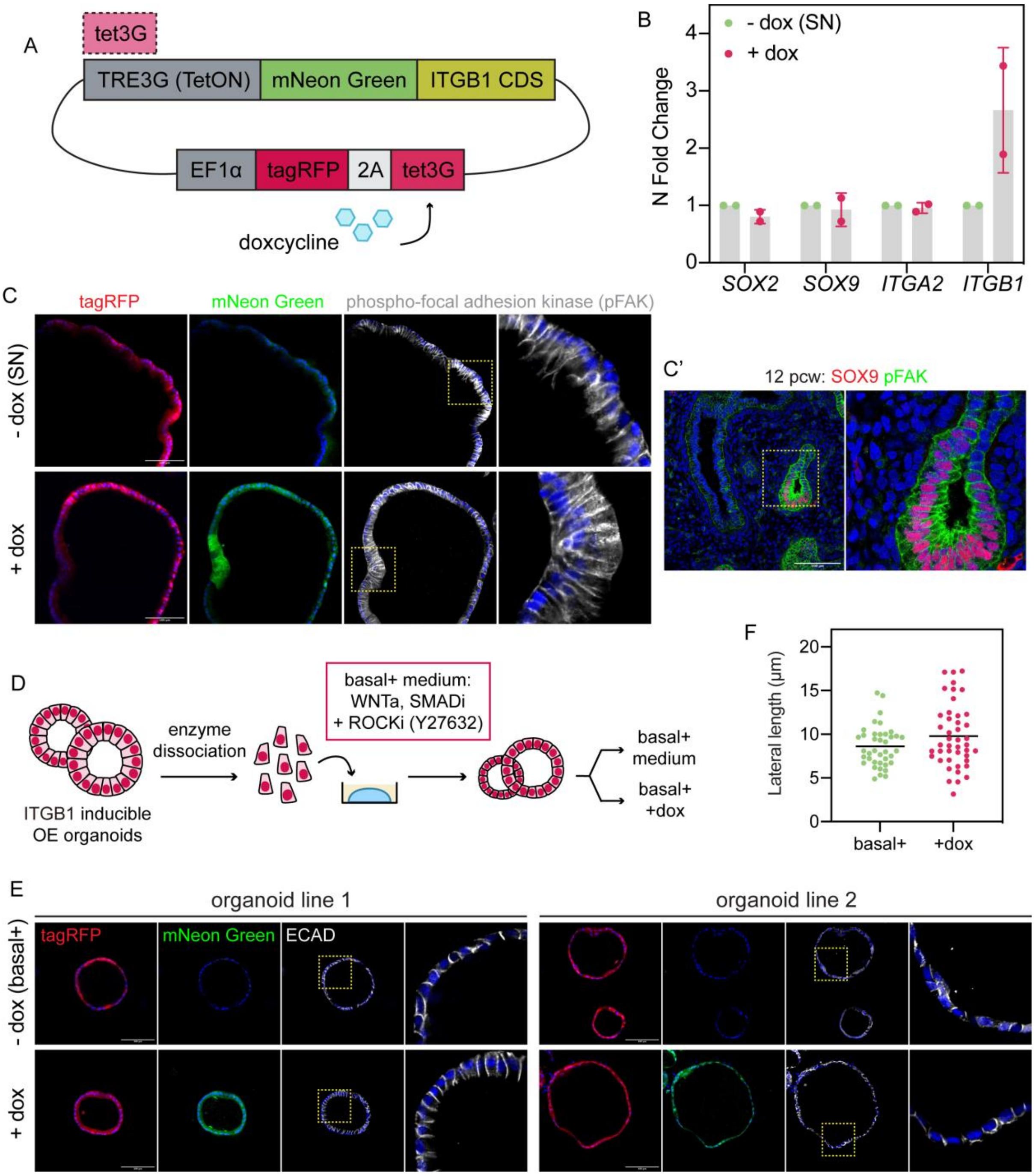

In addition to genetically overexpressing *ITGB1*, we used $MnCl_2$, which was shown to enhance integrin-mediated cell- matrix adhesion (Bazzoni et al., 1995 PMID: 7592728), to test whether it would have any impact on the tip organoids. Shown in Reviewer Figure 5, adding 50 µM $MnCl_2$ to the SN organoids for 5 days did not exert organoid morphology or cell shape change, or obvious alteration in the expression pattern of α2 and β1 integrins. We reasoned that the SN medium was sufficient to maintain proper cell-matrix adhesion and any potential effect of $MnCl_2$ was masked or very mild. However, negative results were also observed when adding 50 µM $MnCl_2$ to the basal spheres for 14 days. As shown in Fig. 6B and C in the ms, inhibiting MAPK/ERK resulted in the downregulation of several integrin genes. Therefore, withdrawal of the RTK inputs would lead to similar disruption. It is likely that $MnCl_2$ did not recover the cell shape in the basal spheres as several integrin genes are likely downregulated.

Taken together (Integrin B1 overexpression and $MnCl_2$ administration) we conclude that integrin signalling is most likely interacting with RTK signalling in the tip epithelial cells to maintain

© 2023. Published by The Company of Biologists under the terms of the Creative Commons Attribution License (https://creativecommons.org/licenses/by/4.0/).

epithelial polarity and adhesion. It should be noted that integrins are regulated at multiple levels, not just gene expression (for example, see review Paul et al., 2015 PMID: 26583903). In the future it will be required to carefully develop experiments to better understand their roles and crosstalk with other cell surface receptors in the lung tip epithelial cells. This was beyond the scope of our project.

(Note we have not included the data shown in Reviewer Figure 4 and 5 in the manuscript as we do not think that the experiments are fully conclusive and the manuscript already contains a lot of data. However, they could be added to an additional supplemental figure if the editor thinks this is a good idea.)

Reviewer Figure 5: Enhancing integrin-mediated cell-matrix adhesion by $MnCl_2$ did not alter cell shape of the tip organoids.
(A) Experimental design: SN organoids were treated with 50 μM $MnCl_2$ for 5 days to enhance potential integrin-mediated cell-matrix adhesion.
(B) Representative images showing cell shape (illustrated by F-actin) and expression of integrin α2 (ITGA2) and integrin β1 (ITGB1) in $MnCl_2$-treated organoids and SN control. Scale bars = 100 μm.
(C) Quantitation of cell lateral length from two organoid lines. Mean±s.d. are shown.
(D) Experimental design: SN organoids were dissociated into single cells, seeded in Matrigel and cultured in the basal+ medium to form basal spheres. The basal spheres were treated with 50 μM $MnCl_2$ for 14 days to enhance potential integrin-mediated cell-matrix adhesion.
(E) Representative images showing cell shape (illustrated by F-actin) of $MnCl_2$-treated organoids and the basal sphere control. Scale bar = 100 μm.

© 2023. Published by The Company of Biologists under the terms of the Creative Commons Attribution License (https://creativecommons.org/licenses/by/4.0/).

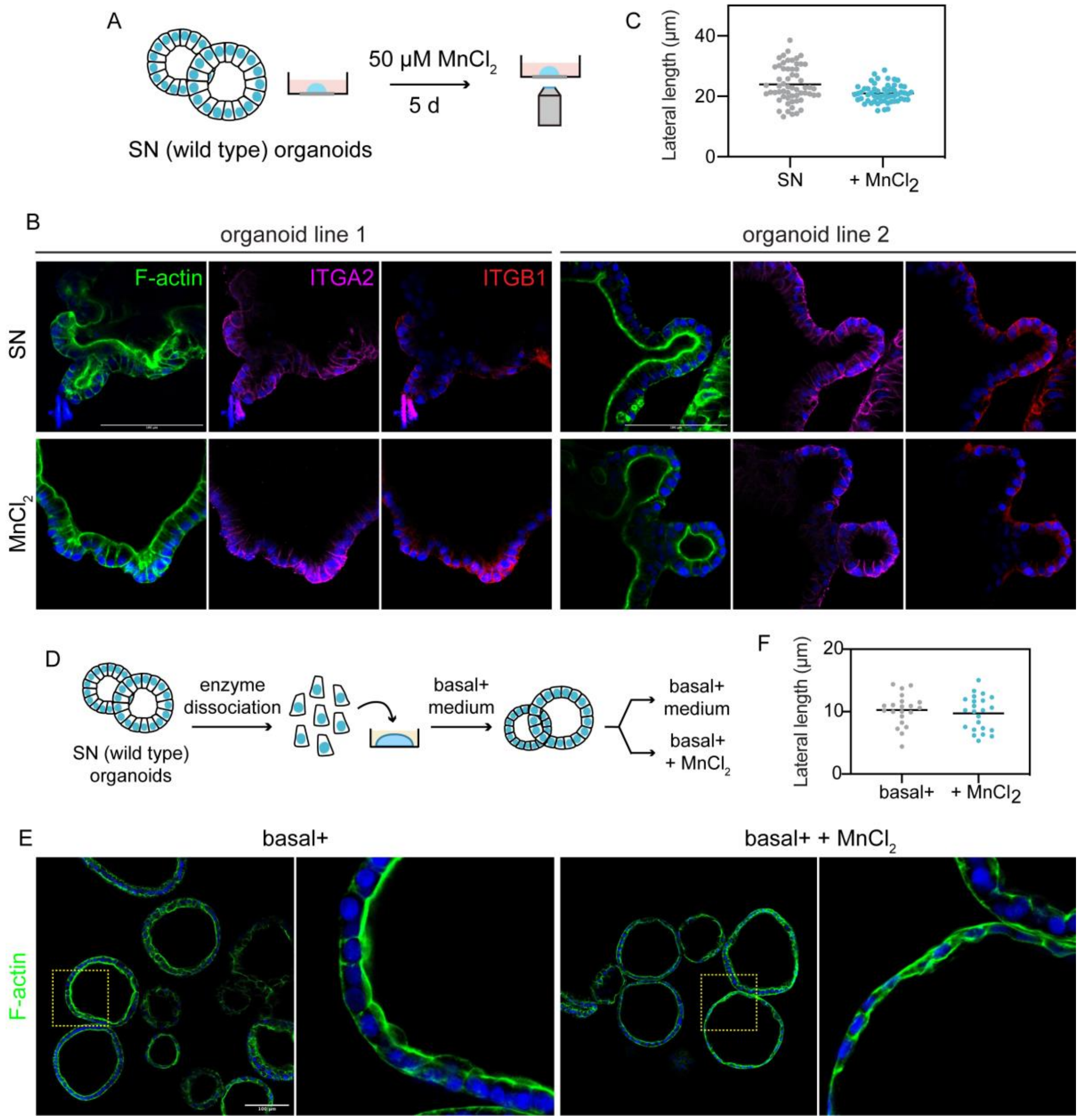

Does knocking down or blocking Itga2 alone affect cell shape? Broadening to Itgb1, a partner for the majority of alpha integrins, when their RNA-seq data implicated specific integrins, weakens their conclusions to the generic “integrin signaling”, which is predictably important. It does not help with the specificity and significance to describe integrins are regulated by MAP/ERK and also regulate MAP/ERK.

We postulate that disrupting ITGA2 alone might exert some mild cell shape changes for two reasons, although we have not performed any experiments. One was that *ITGA2* is the highest expressed alpha integrin gene in the developing human lung tip epithelia (Nikolic et al., 2017 PMID: 28665271). Therefore, downregulating *ITGA2* or functionally blocking it would potentially interfere with integrin α2β1. Together with our findings in this manuscript, we therefore thought the cell shape would be altered consequently. However, there are several other alpha integrin genes expressed in the developing human lung tip epithelia (Nikolic et al., 2017 PMID: 28665271) and it is likely that these could compensate for the downregulation of *ITGA2*. This was also the reason that we blocked both integrin α2, the most abundant integrin alpha subunits and a target of MAPK/ERK in the tip epithelial cells, and integrin β1, the binding partner for the majority of alpha integrins, in order to figure out the roles of integrin signalling rather than any specific isoform.

We agree that integrin signalling is predictably important in the tip epithelial cells, just like many other pathways. However, the gap in knowledge has been the underlying cellular mechanisms. Therefore, we disrupted integrin signalling using antibodies and genetic manipulation, to identify

© 2023. Published by The Company of Biologists under the terms of the Creative Commons Attribution License (https://creativecommons.org/licenses/by/4.0/).

phenotypes at cellular and molecular levels. The RNA-seq data have determined several integrin genes as targets of MAPK/ERK pathway. Interestingly, we have shown that in the lung tip epithelial cells, integrin signalling is also an input into the MAPK/ERK pathway (Fig. 6K). In our opinion, the next step will be to understand how the two pathways interact and to identify potential feedback regulation.

Another example of insufficient understanding of conflicting data is that they spent much of the manuscript on FGF7, contrasting it to FGF10 and EGF and the former's role in the mouse lung. This comparison was made less impactful by the suggestion at the end of the manuscript that FGF10 and EGF are somehow involved for cell shape maintenance.
Our lab has previously published that FGF7, FGF10 and EGF are all required to successfully derive and maintain long- term self-renewing tip epithelial cells from human embryonic lungs at the early pseudoglandular stage (Nikolic et al., 2017 PMID: 28665271). Our recent publication has further confirmed that FGF7, FGF10 and EGF are requested for *in vitro* derivation and expansion of tip epithelial cells from 16 to 22 pcw human embryonic lungs (Lim et al., 2022 PMID: 36493780).

Shown in Fig. 1F, 2B and S13, we found that among FGF7, FGF10 and EGF, it was FGF7 that individually played the most important role in maintaining the tip epithelial cells *in vitro*. However, our understanding of lung development has previously been from mouse work and in the mouse lung, FGF10 is the key FGF ligand. Thus, our observations and the mouse-human species differences prompted us to first focus on understanding FGF7 in the developing human lung.

We came back to FGF10 and EGF at the end of the manuscript for two main reasons. One was that we were confident that both were required *in vitro* for the derivation and self-renewal maintenance of the tip epithelial progenitor cells. Naturally, we would like to explore their roles as much as possible. The other was that we began to realize in the context of this manuscript, that the levels of MAPK/ERK and AKT signalling resulting from multiple RTK inputs were the key to the maintenance of the progenitor cell shape. Thus, we were wondering whether FGF10 and EGF in combination would act differently to individual ligands and increase signalling activation. As mentioned by Reviewer 2 and shown in Fig. 7J, K and new data in Figure S13, FGF10 and EGF showed combinatorial effects on the tip epithelial cells through ERK activation, and the level of the signalling activation may contribute to the columnar cell shape and cell junctions.

In vivo, it is extremely rare to find only one FGF or EGF family member expressed in a specific developmental scenario. We therefore consider that the finding that the cellular phenotypes are a function of the amount of downstream pathway activity elicited by a specific ligand, or ligand combination, is a central finding of the work and will be highly interesting to readers of Development.

Relatedly, they used two organoid culture systems (self-renewing and basal sphere) and two dissociation methods (mechanical and enzymatic) interchangeably, although there are differences including the mentioned conflicting data on FGF10 and EGF. Does gene function depend on culture conditions? Which condition recapitulates gene function in vivo? We would like to first clarify that in both culture systems/passaging methods, our findings on FGF10 and EGF were concordant. Neither FGF10 or EGF alone was capable of maintaining the columnar cell shape and thus cell junctions, whereas the two showed combinatorial effects.

For the two organoid culture systems:

- Basal spheres were typically derived from freshly isolated tip cells throughout the manuscript, including in Figure 7C where the response to FGF10/EGF combined is shown.

- However, basal spheres cannot be maintained long-term and the number of fresh human lungs available is limiting. For some assays it was therefore necessary to derive simple spheres from the self-renewing medium, including for the analysis of FGF10/EGF in Figure 7. (All the western blots also originated from SN organoids as more cells were required than could be obtained from primary tissue).

© 2023. Published by The Company of Biologists under the terms of the Creative Commons Attribution License (https://creativecommons.org/licenses/by/4.0/).

For the two dissociation methods:
- we mechanically passaged SN organoids as our routine culture method.
- and enzymatically dissociated the organoids when we needed single cells for purposes such as sorting and counting cell number.

Both dissociation methods are sufficient to stably maintain organoids in Matrigel using the SN medium as multipotent progenitor epithelial organoids. This is now made clear in the methods.

The organoids behave the same in both passaging conditions and the results with RTK signalling presented do not change with passaging condition. We regard organoids are a useful reductionist tool to assess gene function in as close to an in vivo situation as possible at the moment.

A number of immunostaining images are unconvincing. For example, Fig. 4A, 4B, and 4E. Why is the FGFR2 signal nuclear in Fig. 1G?

For the phospho-ERK and phospho-AKT staining that we presented in Fig. 4, we first validated the antibody on organoids treated with MEK inhibitor or AKT inhibitor (Fig. S8B). Applying the widely-used commercial inhibitors, we observed decreased phospho-ERK or phospho-AKT staining. Thus, we concluded that the staining antibodies were effective and specific.

1. 4A and 4B are the primary human tissue. There is a varying delay between tissue harvest and fixation in all of the human embryo/foetal experiments due to how the tissue bank operates. The phopho-antibodies are likely to be particularly sensitive to this delay. We therefore put images of the most representative pictures in 4A and 4B, but also included all the other staining patterns observed in Fig. S4 to be honest with the reader about the variability.
2. 4E – the pAKT staining is not particularly pretty. However, it was highly reproducible, as can be seen in the quantitation in 4F. And our inhibitor experiments confirm that it is specific.
3. For the nuclear signal FGFR2 in Fig. 1G, we would like to first point out that FGFR2 has previously been detected in the cell nucleus and there is evidence that it can function here to regulate gene expression (see review Tuzon et al., 2019, PMID: 30982184). We think the accurately represented FGFR2 expression in the two 11 pcw samples we used. However, sample-to-sample variation does always exist in humans and we show both replicates for transparency.

Fig. 1H needs single channel images for p-pan FGFR and p-EGFR like 1G. If stronger data is not available, the gap in evidence needs to be stated.

We thank the Reviewer for this comment. We have now shown separate channels in Fig. 1H to make it clearer.

Line 116 says “removing EGF did not cause a striking morphology change (Fig. 1F)”, but the images look otherwise to this reviewer.

The organoids tend to show different morphologies even in the same Matrigel dome. Taking together different biological replicates, we decided not to over-conclude these observations in the EGF-removed medium but describe the phenotype in a milder tone. We agree that the morphology does change, and have altered the text to write: whereas removing EGF did not cause such clear morphology changes (Fig. 1F).

Line 329: “integrin protein was altered (Fig. S11A)”. Could this expression pattern simply be a result of cell shape change and not altered integrin?

We agree that in S11A we cannot distinguish between the integrin expression being altered due to MAPKi, or due to cell shape changes. This is why we simply write that the integrin expression is altered and conclude that, together with the integrin transcriptional changes observed following MAPKi, it is worth investigating the integrin pathway further.

Fig. 4K’ is so variable that it needs statistical testing.

We thank the Reviewer for this comment. We have now performed one-way ANOVA test and added statistical results to Fig. 4K'.

Fig. 6B:
how are the genes selected? If based on differential expression between treatments, clustering by treatment is expected by design. It should be top variable genes across samples without grouping by treatments.

The KEGG pathway analysis in 6C shows all pathways that were obtained. In addition, the full gene list is available as a supplemental table and is already publicly accessible on GEO (GSE211308).

© 2023. Published by The Company of Biologists under the terms of the Creative Commons Attribution License (https://creativecommons.org/licenses/by/4.0/).

In Fig. 6B we chose to show genes that were of most interest to us in the context of this manuscript, and which also met the following criteria: highly expressed, significantly altered, transcription factors, encoding proteins that are components of MAPK/ERK pathway, cytoskeletons, cell-matrix interactions.

Minor points:
Line 30: extra period or typo somewhere.

Corrected

Line 65: “its role in the development” extra “the”

Corrected

Line 179: “a high level of proliferation was required for branching in vivo and organoid budding in vitro”. Should be correlated without evidence for requirement.

Corrected to ‘correlated with’

Line 291: The method here should be transmission electron microscopy (TEM).

Here the reviewer is incorrect. The EM images are taken on an SEM microscope and therefore need to be referred to as SEM images. However, when imaging semi-thin sections the Verios 460 SEM gives almost-TEM quality images. This is all detailed in the methods. We have changed SEM to EM in the main text to avoid confusion for the readers.

Line 297: “apical surface…was stretched” what does stretched mean in this context? How is stretch classified in TEM?

Corrected to elongated (ie longer than the control). We are not measuring stretch.

Line 327: “high abundance in organoids” the figure referenced is of tissue, not organoids.

This was written unclearly and the reference/figure legend moved to clarify.

Line 360: fig. S11C does not appear to be related to the “integrin antibody blocked organoids”

This has been corrected.

Fig. 1B: missing DAPI label

DAPI is blue throughout and listed as such in each figure legend, but only labelled on the figure in 1A.

Fig 1D: typo in “passaing”

Corrected in 1D (and S13A!)

Fig. 3D: first panel is missing outline of closeup area

Added

Fig. 5I: label style between panels does not match (AKTi is black).

Corrected

Fig. S2F says patchy while the main text says partial

© 2023. Published by The Company of Biologists under the terms of the Creative Commons Attribution License (https://creativecommons.org/licenses/by/4.0/).

We think that these words mean the same in the context used.

Second decision letter

MS ID#: DEVELOP/2022/201284

MS TITLE: RTK signalling promotes epithelial columnar cell shape and apical junction maintenance in human lung progenitor cells

AUTHORS: Shuyu Liu, Dawei Sun, Richard Butler, and Emma L Rawlins
ARTICLE TYPE: Research Article

I am happy to tell you that your manuscript has been accepted for publication in Development, pending our standard ethics checks.

Reviewer 1

Advance summary and potential significance to field

The authors have performed additional experiments to satisfactorily address this reviewer's concerns.

Comments for the author

The authors have satisfactorily addressed this reviewer's concerns.

Reviewer 2

Advance summary and potential significance to field

The authors accurately understood and responded to the reviewers' comments.

Comments for the author

This reviewer was fully satisfied with the additional data and answers provided by the authors. The paper is now in good standing and deserves to be published in this journal.

Reviewer 3

Advance summary and potential significance to field

See prior review.

Comments for the author

This reviewer feels it necessary to include the FGFR2 mosaic analysis and ITGB1 rescue experiments in the publication, if they are considered technically sound.

© 2023. Published by The Company of Biologists under the terms of the Creative Commons Attribution License (https://creativecommons.org/licenses/by/4.0/).
